# MicroRNAs regulating superoxide dismutase 2 are new circulating biomarkers of heart failure

**DOI:** 10.1038/s41598-017-15011-6

**Published:** 2017-11-07

**Authors:** Emilie Dubois-Deruy, Marie Cuvelliez, Jan Fiedler, Henri Charrier, Paul Mulder, Eleonore Hebbar, Angelika Pfanne, Olivia Beseme, Maggy Chwastyniak, Philippe Amouyel, Vincent Richard, Christophe Bauters, Thomas Thum, Florence Pinet

**Affiliations:** 1 INSERM, U1167, FHU-REMOD-HF, Institut Pasteur de Lille, University Lille Nord de France, 59800 Lille, France; 2 Institute of Molecular and Translational Therapeutic Strategies (IMTTS), IFB-Tx, Hannover Medical School, Hannover, Germany; 3 Normandie Univ, UNIROUEN, Inserm U1096, FHU-REMOD-HF, 76000 Rouen, France; 4 Centre Hospitalier Régional et Universitaire de Lille, 59800 Lille, France

## Abstract

Although several risk factors such as infarct size have been identified, the progression of heart failure (HF) remains difficult to predict in clinical practice. Using an experimental rat model of post-myocardial infarction (MI), we previously identified 45 proteins differentially modulated during HF by proteomic analysis. This study sought to identify microRNAs (miRNAs) able to regulate these proteins and to test their relevance as biomarkers for HF. *In silico* bioinformatical analysis selected 13 miRNAs related to the 45 proteins previously identified. These miRNAs were analyzed in the rat and in cohorts of patients phenotyped for left ventricular remodeling (LVR). We identified that 3 miRNAs, miR-21-5p, miR-23a-3p and miR-222-3p, and their target Mn superoxide dismutase (SOD2) were significantly increased in LV and plasma of HF-rats. We found by luciferase activity a direct interaction of miR-222-3p with 3′UTR of SOD2. Transfection of human cardiomyocytes with miR-222-3p mimic or inhibitor induced respectively a decrease and an increase of SOD2 expression. Circulating levels of the 3 miRNAs and their target SOD2 were associated with high LVR post-MI in REVE-2 patients. We demonstrated for the first time the potential of microRNAs regulating SOD2 as new circulating biomarkers of HF.

## Introduction

Chronic heart failure (HF) remains a major cause of illness and death and its prevalence is increasing with a high rate of morbidity and mortality^1^. Despite major significant advances, HF remains a therapeutic challenge, and several adverse consequences of HF are still poorly controlled. New prognostic or diagnostic biomarkers of HF are still important to find and proteomic approaches associated with bioinformatic tools may be useful^2^.

Novel determinants of post-myocardial infarction (MI)-HF were already revealed by previous proteomic^3^ and phosphoproteomic^4^ approaches using an experimental rat model of HF. We especially discovered that HF is associated with decreased levels of serine^208^-phosphorylated troponin T (TnTpS208) in left ventricle (LV) and plasma of HF-rats. The same decrease was found in patients with high left ventricular remodeling (LVR) after MI, suggesting that the level of circulating TnTpS208 could be a new biomarker of LVR and may help to predict the development of HF after MI^5^.

MicroRNAs (miRNAs) are non-coding RNAs identified as regulatory molecules consisting of 19 to 23 nucleotides that regulate gene expression by hybridization to messenger RNAs with the consequence of its degradation or translational inhibition of targeted transcripts^6^. miRNAs are highly conserved and play a role in many biological processes such as cell-cell communication^7^ or signalling pathways^8^. miRNAs, some with a tissue- and pathology-specific expression, could be involved in several cardiovascular pathologies such as cardiac hypertrophy^9,10^, adverse cardiac remodeling^11,12^ or HF^11,13^. miRNAs can be secreted in either protein-bound or vesicle-enclosed forms from the cells into the circulation, suggesting their potential as biomarkers in molecular diagnostics^13^. These previous studies suggest that levels of circulating miRNAs may depend on several aspects not directly related to disease states, such as miRNA tissue specificity, mechanisms of release and retention, mechanisms of transport onto the bloodstream and pathways of degradation.

Up to now, we failed to identify miRNAs, such as miR-133a and miR-423-5p, as circulating prognostic biomarkers of cardiac remodelling post-MI^14^. Our strategy was to use bioinformatics tools such as Ingenuity Pathway Analysis (IPA) to integrate our proteomic data obtained in the experimental rat model of HF in order to decipher miRNAs interacting directly or indirectly with the 45 proteins found to be differentially modulated. Interestingly, we evidenced 17 proteins involved in oxidative stress and metabolism^3^. Among them, we focused on superoxide dismutase [Mn], mitochondrial (SOD2), a major antioxidant enzyme, notably described for protecting the morphology of the diabetic heart and completely normalizing contractility in diabetic cardiomyocytes^15^.

The purpose of this work was i) to select by bioinformatics miRNAs interacting with proteins identified by proteomic analysis to be modulated by HF in the rat model, ii) to validate the LV expression and modulation of these selected miRNAs, iii) to determine whether circulating plasma levels of miRNAs match with heart specificity in order to improve our understanding of relationship between LV tissue levels and plasma levels; iv) to characterize how they affect SOD2 expression in cardiomyocytes and, v) to confirm their potential as biomarkers of cardiac remodeling by quantification of circulating plasma levels of the miRNAs and SOD2 in human patients phenotyped for LV remodeling.

## Results

### Identification of candidate miRNAs for heart failure

A total of 63 rats were included in the analysis; 15 were sacrificed 7 days after surgery (8 sham and 7MI) and 48 were sacrificed 2 months after surgery (25 sham and 23 MI). Detailed echocardiographic, hemodynamic and morphometric parameters of sham- and MI-rats are provided (Supplementary Table 1). Briefly, 7 days after MI, a significant increase in heart weight was already observed. After 2 months, MI induced classic signs of HF, i.e. significant increases in LV diameters and LV end-diastolic pressure, as well as marked decreases in LV fractional shortening, LV dP/dt and cardiac output, associated with significant cardiac hypertrophy.

**Table 1:** Characteristics of the patients included in the REVE-2 study (n = 246).

Characteristics	All	men	Women
n	246	200	46
Age, yr (mean ± SD)	57 ± 14	56 ± 13	62 ± 15
Diabetes mellitus	51 (21%)	46 (23%)	5 (11%)
Initial reperfusion therapy			
Primary percutaneous coronary intervention	128 (52%)	106 (43%)	22 (48%)
Thombolysis alone	28 (11%)	23 (9%)	5 (11%)
Thombolysis and rescue percutaneous intervention	59 (24%)	50 (20%)	9 (20%)
No reperfusion	31 (13%)	21 (11%)	10 (22%)
Peak creatitine kinase, IU (mean ± SD)	3018 ± 2376	3099 ± 2483	2666 ± 1824
HF (Killip class ≥2) during hospitalization	79 (32%)	65 (33%)	14 (30%)
LVEF, % (mean ± SD)	49 ± 8	49 ± 8	51 ± 9
Medications at discharge			
Antiplatelet therapy	246 (100%)	200 (100%)	46 (100%)
β-blokers	238 (97%)	194 (97%)	44 (96%)
ACE inhibitors	238 (97%)	193 (97%)	45 (98%)
Statins	231 (94%)	189 (95%)	42 (91%)
One-year echocardiography follow-up			
Number of patients with follow-up	223 (92%)	182 (91%)	44 (96%)
LV remodeling*, n	87 (38.5%#)	67 (36.8%#)	20 (45.5%#)

ACE indicates angiotensin-converting enzyme; HF, heart failure; IU, International units; LV, left ventricular; LVEDV, left ventricular end-diastolic volume; and LVEF, left ventricular ejection fraction.*Defined as a >20% change in LVEDV between baseline and 12 months. #Of the patients with echocardiographic follow-up.

**Figure 1:**
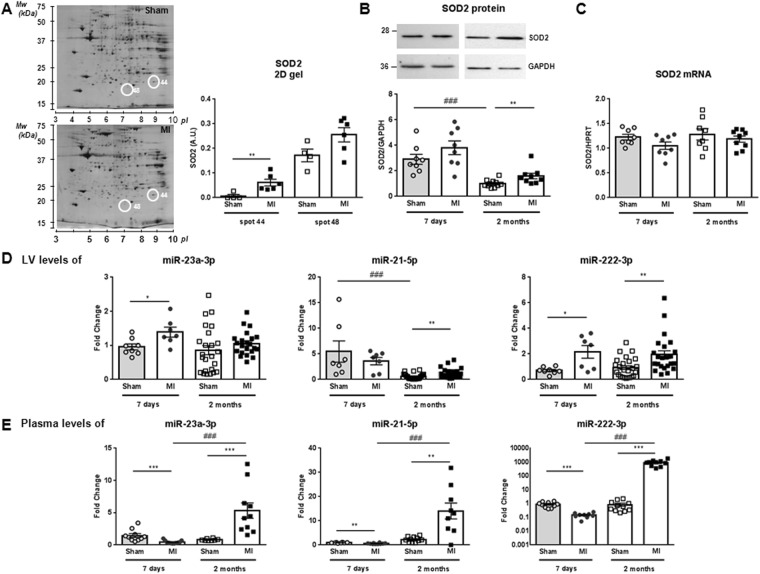
SOD2 and miRNAs expression in LV and plasma of MI-rats. (**A**) Representative 2D-gel of rat LV proteins from sham and 2-months MI-rats (left panel). The positions of molecular weight (Mw) are indicated on the left and the *pI* on the bottom of the gels. Spots identified by mass spectrometry as SOD2 are encircled. Spots number correspond to proteomic analysis previously published^3^. Quantification of the 2 spots corresponding to SOD2 was obtained from 4 sham- and 6 MI-rats (right panel). (**B**) Representative western blot of SOD2 protein in LV extracted from rat 7 days (circle, n = 8) and 2 months (square, n = 8) after MI (n = 16) or sham (n = 16) surgery. Data were normalized to GAPDH for western blot. (**C**) RT-qPCR of SOD2 mRNA in LV extracted from rat 7 days (circle, n = 8) and 2 months (square, n = 8) after MI (n = 16) or sham surgery (n = 16). Data were normalized to HPRT for RT-qPCR. (**D**) RT-qPCR of miR-21-5p, miR-23a-3p and miR-222-3p in LV extracted from rat 7 days (circle, n = 8) and 2 months (square, n = 8) after MI (n = 16) or sham (n = 16) surgery. Data were normalized to miR-423-3p for LV and graph shows mean ± SEM values expressed as fold change (2−ΔΔCt). (**E**) RT-qPCR of miR-21-5p, miR-23a-3p and miR-222-3p in plasma extracted from rat 7 days (circle, n = 9) and 2 months (square, n = 9) after MI (n = 18) or sham (n = 18) surgery. Data were normalized to Cel-39 for plasma and graph shows mean ± SEM values expressed as fold change (2^−ΔΔ*C*t^). *p < 0.05; **p < 0.01; ***p < 0.001, for 2 groups’ comparison (nonparametric Mann–Whitney test) and ###p < 0.001, for multiple groups’ comparison (Kruskall-Wallis with Dunn’s multiple comparison test).

We previously identified by proteomic analysis and mass spectrometry 26 proteins differentially expressed^3^ (Fig. 1A) and 30 proteins differentially phosphorylated^4^ in LV of 2 months HF-rats. These proteins were overlaid onto a global molecular network in the IPA Knowledge Base in order to select miRNAs interacting with the proteins identified by proteomic analysis (Supplementary Table 2). IPA analysis identified interactions between 8 proteins and 13 miRNAs (Supplementary Table 2). We found a significant increase in SOD2 protein in LV (Fig. 1B) but no modulation of SOD2 mRNA (Fig. 1C). Interestingly, SOD2 is regulated by 5 of 13 miRNAs selected by IPA, i.e. mir-21-3p, miR-21-5p, miR-23a-3p, miR-145-5p and miR-222-3p (Supplementary Fig. 1A).

We quantified expression of the 13 miRNAs in LV of 7 days and 2 months MI-rats. Unfortunately, we could not deepen the cluster between peroxiredoxin-2, protein disulfide isomerase and the miR-122-5p and miR-210 due to a lack of specificity of different primers tested (not shown). Among the 11 miRNAs quantified, 4 were not modulated after 7 days or 2 months MI: miR-29b-3p, miR-338-3p, miR-133a and miR-483-3p interacting respectively with tropomyosin alpha-1 chain, pyruvate kinase PKM and phosphoglycerate mutase 1 (Supplementary Fig. 1B–E). Moreover, we observed a significant increase in miR-320a and in miR-377-5p in LV of HF-rats respectively in 7 days and 2 months MI-rats (Supplementary Fig. 1F–G).

### Post-transcriptional regulators of SOD2 expression

First, we quantified the 5 miRNAs identified to be associated to SOD2 by IPA analysis. Expression of miR-145-5p was significantly increased in 2 months-rats compared to 7 days-rats (Supplementary Fig. 1A). We observed a significantly increased expression of the 4 other miRNAs in LV, at 7 days post-MI for miR-23a-3p (Fig. 1D), at 2 months post-MI for miR-21-5p (Fig. 1D) and miR-21-3p (Supplementary Fig. 1A) and at both times for miR-222-3p (Fig. 1D).

We next quantified these 4 miRNAs in plasma of the same animals. We were unable to quantify detectable levels of miR-21-3p. Interestingly, miR-21-5p, miR-23a-3p and miR-222-3p were significantly decreased in the plasma of 7 days MI-rats and significantly increased at 2 months post-MI (Fig. 1E) without any modulation of SOD2 in plasma of HF-rats (not shown).

### Role of SOD2 and its post-transcriptional regulators in cardiac hypertrophy

**Figure 2:**
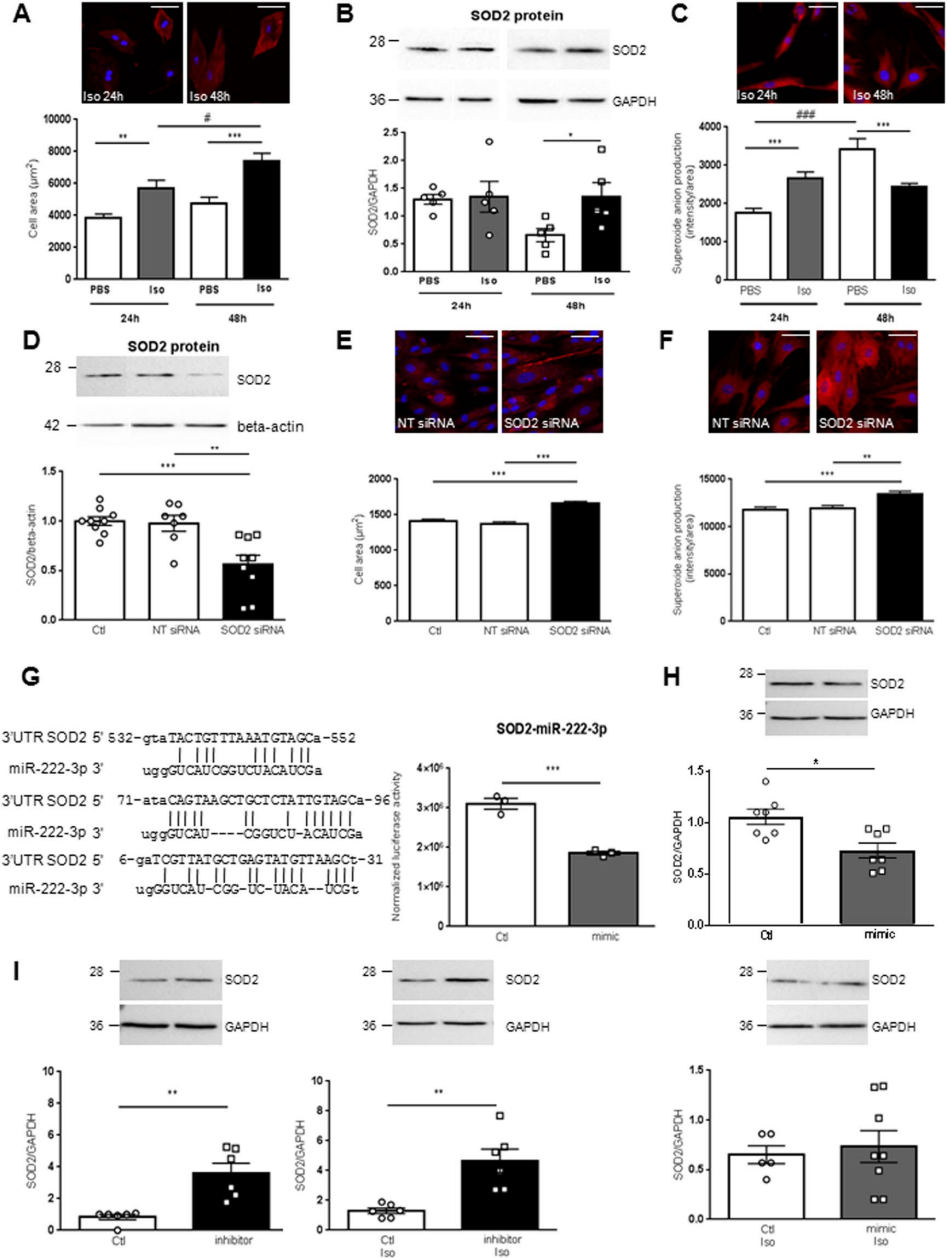
Role of SOD2 and its post-transcriptional regulators in cardiac hypertrophy. (**A**) Representative immunofluorescence of alpha-actinin (red) in H9c2 cardiomyoblasts Iso treated for 24 h and 48 h (top panel). Quantification after 24 h and 48 h of cell area was performed from 3 independent experiments and at least 28 cells untreated (PBS) or Iso treated. Graph shows mean ± SEM values expressed as cell area (µm^2^) (lower panel). (**B**) Representative western blot of SOD2 protein in H9c2 cardiomyoblasts untreated or Iso treated for 24 h and 48 h (top panel). Quantification was performed from at least 4 independent experiments. Data were normalized to GAPDH and graph shows mean ± SEM values expressed as SOD2/GADPH (lower panel). (**C**) Representative immunofluorescence of mitoSOX (red) in H9c2 cardiomyoblasts Iso treated for 24 h and 48 h. Mitochondrial superoxide anion production was quantified from 3 independent experiments and at least 60 cells. Graph shows mean ± SEM values expressed as superoxide anion production intensity/cell area (lower panel). (**D**) Representative western blot of SOD2 protein in H9c2 cardiomyoblasts control (Ctl: only DharmaFECT® reagent) or transfected with non-target (NT siRNA) or SOD2 siRNA for 72 h (top panel). Quantification of SOD2 was performed from at least 5 independent experiments. Data were normalized to beta-actin and graph shows mean ± SEM values expressed as SOD2/beta-actin (lower panel). (**E**) Representative immunofluorescence of alpha-actinin (red) performed in H9c2 cardiomyoblasts Ctl or transfected with NT or SOD2 siRNA for 72 h (top panel). Quantification of cell area was performed from 3 independent experiments and at least 193 cells. Red intensity measured for each cell was normalized to the cell area and graph shows mean ± SEM values expressed as cell area (µm^2^) (lower panel). (**F**) Representative immunofluorescence of mitoSOX (red) performed in H9c2 cardiomyoblasts Ctl or transfected with NT or SOD2 siRNA for 72 h (top panel). Mitochondrial superoxide anion production was quantified from 3 independent experiments and at least 182 cells. Red intensity measured for each cell was normalized to the cell area and graph shows mean ± SEM values expressed as superoxide anion production intensity/cell area (lower panel). Nuclei were stained with Hoechst (blue). Scale bar is 50 µm (A, C, E, F). (**G**) Sequence of the three binding sites of miR-222-3p identified on human 3′-UTR SOD2 with miRTarBase (Release 6.0: Sept. 15, 2015) (left panel). Quantification of luciferase activity in HEK293 cells after transfection of control (white) and miR-222-3p mimic (grey) (right panel). Data were obtained from 3 independent experiments and graph shows mean ± SEM values expressed as normalized luciferase acivity. (**H**) Representative western blot of SOD2 in human cardiomyocytes either control (white) or transfected by miR-222-3p mimic (grey) in untreated (top panel) or treated with Iso for 48 h (lower panel). Quantification of SOD2 in human cardiomyocytes was obtained from 4 independent experiments. Data were normalized to GAPDH and graph shows mean ± SEM values expressed as SOD2/GADPH. (**I**) Representative western blot of SOD2 in human cardiomyocytes either control (white) or transfected by miR-222-3p inhibitor (black) in untreated (left panel) or treated with Iso for 48 h (right panel). Quantification of SOD2 in human cardiomyocytes was obtained from 4 independent experiments. Data were normalized to GAPDH and graph shows mean ± SEM values expressed as SOD2/GADPH *p < 0.05; **p < 0.01; ***p < 0.001.

We investigated the role of SOD2 during cardiac hypertrophy in H9c2 cells (Fig. 2A–C). First, we validated that Isoproterenol (Iso) (10 µM) induces hypertrophy verified by the significant increased cell area at both 24 h and 48 h (Fig. 2A). Interestingly, we observed a significant increase in SOD2 protein expression only after 48 h Iso (Fig. 2B), despite no modulation of SOD2 mRNA or of the 3 miRNAs (data not shown). With MitoSOX probe, we observed a significant increase in mitochondrial superoxide anion levels quantified after 24 h of hypertrophy as expected. On the contrary, after 48 h Iso, superoxide anion production was significantly decreased (Fig. 2C), validating an activation of SOD2 at this time.

We then analyzed the role of SOD2 on hypertrophy and oxidative stress by silencing it in H9c2 cells (Fig. 2D–F). First, we validated that SOD2 siRNA transfected in H9c2 cells induced a significant decrease in SOD2 protein expression (Fig. 2D). Interestingly, we observed that SOD2 silencing induced significantly hypertrophy and oxidative stress quantified respectively, by the cell area (Fig. 2E) and the mitochondrial superoxide anion levels (Fig. 2F).

### miR-222-3p is a post-transcriptional regulator of SOD2

Among the 3 miRNAs identified to interact with SOD2 in our experimental rat model of HF, only miR-222-3p was predicted to directly interact with SOD2 in human (Fig. 2G). To confirm this interaction, we cloned human SOD2 3′UTR (untranslated region) harboring two potential binding sites for miR-222-3p in HEK293 cells. Cotransfection of miR-222-3p mimic with human SOD2 3′UTR induced a significant decrease in luciferase expression, suggesting that miR-222-3p binds to SOD2 3′UTR (Fig. 2G).

We then used miR-222-3p mimic and inhibitor to analyze the impact of miR-222-3p on SOD2 expression in human Cytivia plus cardiomyocytes. We first validated the efficiency of transfection of miR-222-3p mimic and inhibitor (not shown). We observed that miR-222-3p mimic induced a significant decrease in SOD2 protein expression (Fig. 2H, top panel) counteracted by Iso treatment (Fig. 2H, lower panel). Conversely, miR-222-3p inhibitor induced a significant increase in SOD2 protein expression (Fig. 2I, left panel) not affected by Iso treatment (Fig. 2I, right panel).

### Circulating miRNAs interacting with SOD2 as prognostic biomarkers of HF

Our studies based on *in vivo* rat experimental model and *in vitro* cardiomyocyte models prompted us to assess levels of circulating SOD2 and interacting miRNAS (miR-21-5p, miR-23a-3p and miR-222-3p) in patients with high LV remodeling following MI.

For that purpose, we used blood samples obtained in REVE-2 (“REmodelage VEntriculaire”−2) study^16^. REVE-2 was a multicenter study that enrolled 246 patients with a first anterior wall Q-wave MI followed for one year with serial echographic studies and blood sampling at hospital discharge (baseline) then 3 months and 1 year after MI. REVE-2 patients were divided as no remodeling (LVR < 20%) and high remodeling (LVR > 20%) patients according to the LV remodeling determined as ((EDV1year – EDVbaseline)/EDVbaseline). Baseline characteristics of REVE-2 patients are described in Table 1.

**Figure 3:**
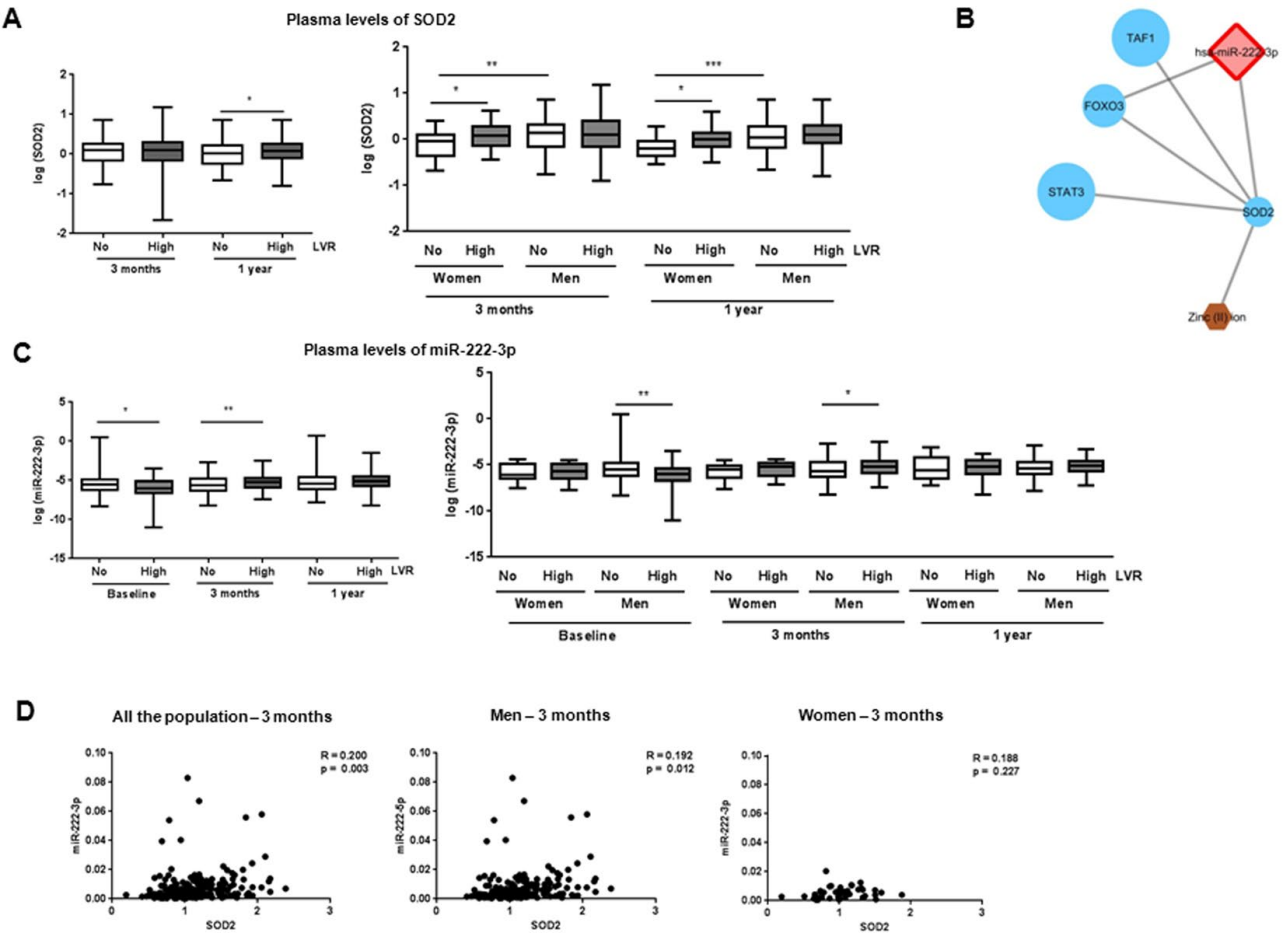
Circulating SOD2 and miRNAs plasma levels of REVE-2 patients. REVE-2 was a multicenter study that enrolled 246 patients with anterior wall Q-wave MI followed for one year with serial echographic studies at hospital discharge (day 3 to day 7) then 3 months and 1 year after MI and serial blood sampling at hospital discharge then 1 month, 3 months, and 1 year after MI. REVE-2 patients (n = 224) were divided as no remodelers (LVR <20%, n = 138)) (white) and high remodelers (LVR > 20%, n = 86) (grey), according to the LV remodeling determined as ((EDV1year − EDVbaseline)/EDVbaseline). (**A**) Quantification by ELISA of SOD2 in plasma from no LV remodeling (white, n = 138) and high LV remodeling (grey, n = 86) patients after 3 months and 1 year post-MI for all the population (left panel). Analysis were also performed by separating women (n = 46, 24 no LVR and 20 high LVR) and men (n = 200, 114 with no LVR and 66 with LVR) (right panel). Data are expressed as log (SOD2) and are represented in box plot with the central rectangle for the interquartile range, the segment inside the rectangle for the median and the segment outside for the minimum and maximum. (**B**) Visualization of the REVE-2 molecular network model^17^ at baseline centralized on direct miR-222-3p interaction. Detailed information are provided as Fig. 2 and Supplementary Table 3. (**C**) Quantification by RT-qPCR of miR-222-3p in plasma extracted from REVE-2 patients at baseline and after 3 months and 1 year post-MI for all the population (left panel). Analysis were also performed by separating women (n = 46, 24 no LVR and 20 high LVR) and men (n = 200, 114 with no LVR and 66 with LVR) (right panel). Data are expressed as log (miR/cel39) and are represented in box plot with the central rectangle for the interquartile range, the segment inside the rectangle for the median and the segment outside for the minimum and maximum. *p < 0.05; **p < 0.01 compared to no LV remodeling patients. (**D**) Pearson correlations between SOD2 and miR-222-3p in REVE-2 patients. The scatter plots show the correlation between SOD2 and miR-222-3p plasma levels at 3 month post-MI for all REVE-2 population (n = 246) (left panel), for men (n = 200) (middle panel) and women (n = 46)(right panel).

SOD2 was quantified in the plasma of REVE-2 patients at 3 months and 1 year post-MI (Fig. 3). We found a significant increase in patients with high remodeling only at 1 year post-MI (Fig. 3A, left panel). We then quantified the 3 miRNAs predicted by IPA to interact with SOD2 (Supplementary Table 2). Interestingly, the REVE-2 molecular network previously built with 23 circulating molecules quantified in blood samples of REVE-2 patients, shows that these 3 miRNAs were highly central molecules with the highest betweeness centrality for miR-21-5p and miR-222-3p^17^, indicating that they are crucial molecules to maintain functionality and coherence of signaling mechanisms in the REVE-2 network. We also identified direct interaction between miR-222-3p, SOD2 and other molecules in the REVE-2 network at baseline (Fig. 3B, details are provided Supplementary Fig. 2 and Supplementary Table 3). The 3 miRNAs were then quantified at baseline (i.e. hospital discharge), 3 months and 1 year post-MI in REVE-2 patients. We found a significant decrease in plasma levels of miR-222-3p (Fig. 3C, left panel), miR-21-5p and miR-23a-3p Fig. 3) at baseline (i.e. hospital discharge) in patients with high remodeling compared to low remodeling following MI. Conversely, we observed a significant increase of the 3 miRNAs in patients with high remodeling at 3 months post-MI and no modulation at 1 year post-MI (Fig. 3C, left panel and Supplementary Fig. 3).

To understand why the levels of the 3 miRNAS and their target, SOD2 are modulated in the same way, we analyzed deeper the REVE-2 molecular network and found an interaction of miR-222-3p with the estrogen receptor 1, located in cluster 22 and characterized by a high betweeness (0.0096), indicating a potential central role of estrogen receptor with miR-222-3p in the REVE-2 network (Supplementary Table 3). Interestingly, the analysis taking into account the gender in REVE-2 population showed significant higher levels of SOD2 in men compared to women (Fig. 3A, right panel). Interestingly, the significant modulation of circulating levels of SOD2 is restricted to women with high remodeling at 3 months and 1 year (Fig. 3A, right panel). Conversely, the significant increase of circulating levels of miR-222-3p is only found in men at 3 months post-MI (Fig. 3C, right panel). The same information was found for the circulating levels of miR-21-5p (Supplementary Fig. 3A, right panel). Moreover, we observed a significant correlation between circulating levels of SOD2 and of miR-222-3p quantified at 3 months post-MI in the whole REVE-2 population and men population but not in women population (Fig. 3D). The same information was found for the circulating levels of miR-21-5p (Fig. 3A, bottom panel) and miR-23a-3p (Supplementary Fig. 3B, bottom panel).

## Discussion

We used a well-characterized model of HF-rat, together with echocardiographic and hemodynamic measurements to identify 45 proteins differentially modulated by HF (e.g. expression and/or phosphorylation levels)^3,4^. Using IPA tools, we selected miRNAs interacting directly or indirectly with these 45 proteins. Indeed, miRNAs are small non-coding RNAs that control various biological processes through affecting the stability and translation of targets mRNAs. IPA analysis identified interactions between 8 proteins and 13 miRNAs. Interestingly, SOD2 is regulated by 5 of the 13 miRNAs.

SOD2 is a well-known antioxidant enzyme, which binds to the superoxide anions to convert them to hydrogen peroxide and oxygen. An increase in SOD2 expression could be beneficial by decreasing reactive oxygen species production as it was described in other diseases such as diabetes. In this pathology, SOD2 protects the morphology of the heart and completely normalizes contractility in cardiomyocytes^15^. Using an *in vitro* model of hypertrophied cardiomyocytes, we found an increase of SOD2 expression associated with decrease of superoxide anion production. Conversely, silencing SOD2 in this model induced hypertrophy and increased superoxide anion production. Despite a high increase in oxidative stress after MI, the role of SOD2 and its post-transcriptional regulators in LVR and HF are still unknown. Here, we focused on 3 miRNAs, miR-21-5p, miR-23a-3p and miR-222-3p and their target SOD2, detected in plasma that we characterized for their potential as biomarkers of HF.

Little is known about the role of miR-23a in cardiovascular diseases, with the exception of a correlation between miR-23a and pulmonary function of patients with idiopathic pulmonary hypertension^18^. In our study, miR-23a-3p increased in LV of HF-rats only at 7 days post MI. miR-21 is highly expressed in most of the cardiovascular cells, especially cardiomyocytes and cardiac fibroblasts^7^. In this study, we observed an increase in miR-21-5p in HF-rats, in accordance with previous reports showing an upregulation of miR-21 in HF with preserved LV ejection fraction^19^ or in fibroblasts of hypertrophied hearts^20^. In most of these studies, miR-21 is associated with an anti-apoptotic effect^19,21^.

MiR-222 has been described to induce cardiomyocytes proliferation and hypertrophy and inhibit apoptosis^22^. Here, we quantified an increase in miR-222-3p expression in HF-rats, in accordance with a previous study showing an increase in miR-222 during physiological hypertrophy at cell and plasma levels^22^. Our *in silico* analysis suggested that only miR-222-3p directly interacts with the 3′-UTR of SOD2. This interaction was already described in oral tongue squamous cell carcinoma cell line^23^. We confirmed this interaction using luciferase reporter gene assays and transfection of miR-222-3p mimic and inhibitor in human cardiomyocytes.

Despite tissue- and pathology-specificity of miRNAs expression, we failed to identify miRNAs, such as miR-133a and miR-423-5p, as circulating prognostic biomarkers of cardiac remodelling post-MI^14^. Another class of non-coding RNAs, long noncoding RNAs (lncRNAs) has been tested successfully in REVE-2 study. We recently described a downregulation of a mitochondrial lncRNA uc022bqs.1, called LIPCAR, early after MI and an upregulation during later stages^24^. LIPCAR is a novel biomarker of cardiac remodeling and predict future death in patients with HF^24^, suggesting potential interest of non-coding RNAs as biomarkers in molecular diagnostics. SOD2 mRNA was recently reported for predicting worse prognosis of patients with untreated classical Hodgkin lymphoma^25^.

Interestingly, by analysing separately women and men in REVE-2 population, we observed that the increase in circulating levels of miRNAs was only significant in men whereas the circulating levels of SOD2 were only increased significantly in women. Our results were supported by previous data showing that estradiol (E2) treatment significantly increases the expression of SOD2 both in mice and in human aorta endothelial cells leading to a decrease in oxidative stress^26^. This could explain why we did not observe any plasmatic modulation of SOD2 in the rat MI model as only male were used.

In this study, we showed for the first time, that levels of circulating miR-21-5p, miR-23a-3p and miR-222-3p decrease in patients with high remodeling at baseline and increase at 3 months post-MI. Our data shows the potential of miR-21-5p, miR23a-3p and miR-222-3p, and their target SOD2, as new biomarkers of post-MI HF.

### Conclusion and perspectives

**Figure 4:**
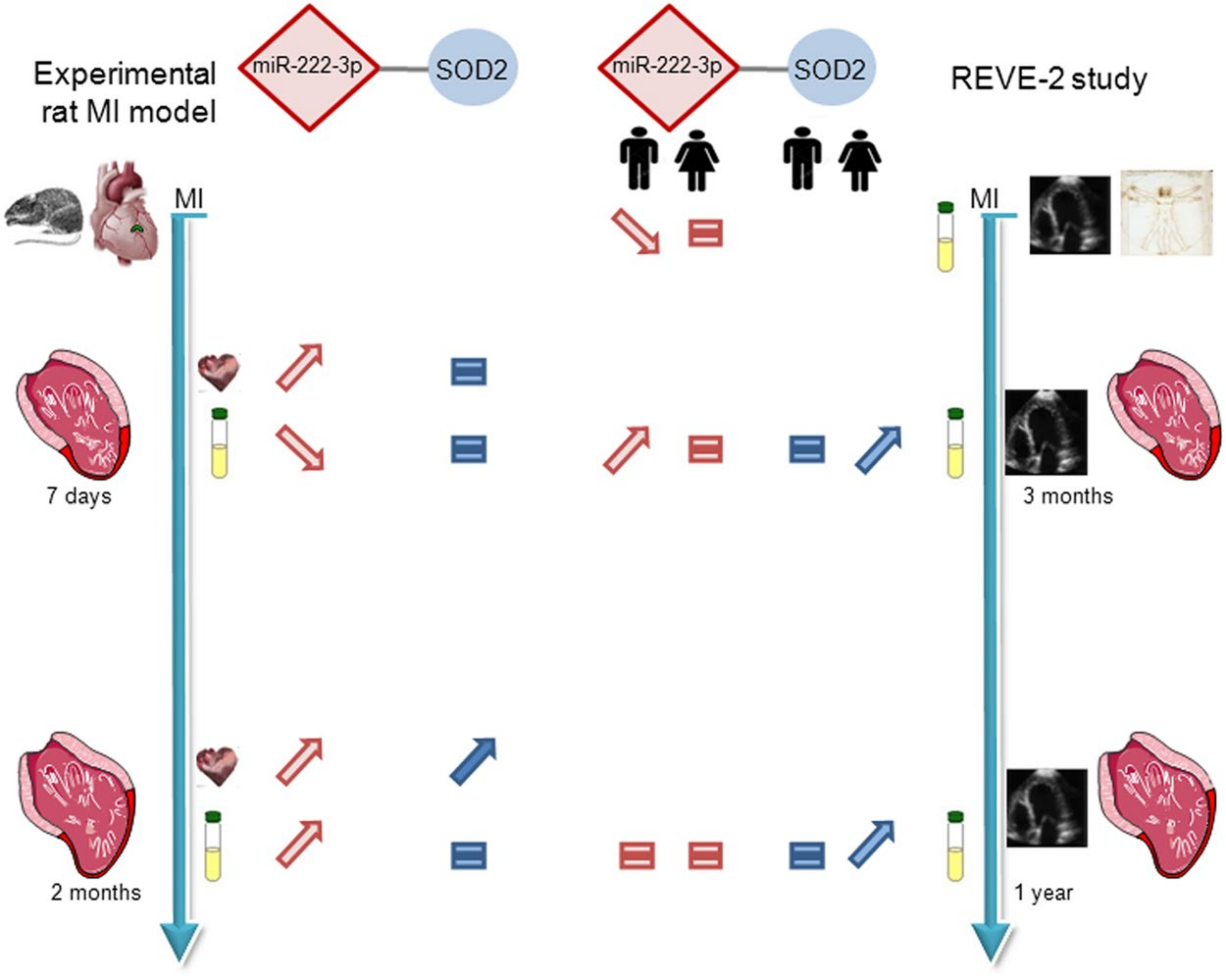
Summary of expression of miR-222-3p and one of its targets SOD2. miR-222-3p and SOD2 were quantified in LV and plasma of experimental model of MI rat at 7 days (n = 9) and 2 months (n = 9) after MI (n = 18) or sham (n = 18) surgery. The circulating levels of miR-222-3p and SOD2 were quantified in plasma of REVE-2 patients (n = 224) respectively at baseline, 3 months, 1 year post-MI and 3 months and 1 year post-MI. Analysis were performed by separating women (n = 46, 24 no LVR and 20 high LVR) and men (n = 200, 114 with no LVR and 66 with LVR).

In this study, we found i) the modulation of 3 miRNAs selected in silico to interact with SOD2 in LV and plasma of the experimental rat model of HF and a significant increase of SOD2 in LV of 2 months post-MI rats; ii) that the circulating levels of the 3 miRNAs were differentially modulated compared to LV at 7 days post-MI, suggesting that plasma expression does not completely reflect the heart specificity at early phase of LV remodeling; iii) how miR-222-3p modulated SOD2 in hypertrophied cardiomyocytes and iv) confirmed their potential as biomarkers of cardiac remodeling in human patients phenotyped for LV remodeling post-MI with gender specificity shown by significant modulation of miR-222-3p in men and of SOD2 in women (Fig. 4). Future works need to deepen the role of SOD2 regulation, notably by the estrogen receptors, and the mechanisms of transport of miRNAs from heart onto bloodstream in the pathophysiology of LVR and HF.

## Methods

A detailed methods section is available as supplemental. All methods were carried out in accordance with relevant guidelines and regulations and all experimental and ethic protocols were approved by Inserm.

### Animal models

Animals were used and experimental protocols performed under the supervision of a person authorized to perform experiments on live animals (F. Pinet: 59-350126 and E. Dubois-Deruy: 59-350253). Approval was granted by the institutional ethics review board (CEEA Nord Pas-de-Calais N°242011, January 2012). MI was induced in 10-week-old male Wistar rats (n = 63) (Janvier, Le Genest St isle, France) by ligation of the left anterior descending coronary artery^27,28^. Haemodynamic and echocardiographic measurements (Supplementary Table 1) were taken 7 days and 2 months after surgery, followed by heart excision and plasma sampling, as previously described^4,29^.

### Bioinformatical analysis

Differentially regulated proteins previously identified by mass spectrometry^3,4^ were further analyzed using Ingenuity Pathway Analysis (IPA, www.ingenuity;com, Winter Release 2012, Ingenuity Systems, Mountain View, CA) (Supplementary Table 2). Networks of these focus proteins were algorithmically generated by including as many focus proteins as possible and other non-focus proteins from the database that are needed to generate the network based on connectivity. Here, we focused our analysis on miRNAs with high predicted and experimentally observed selection by IPA.

### Human samples

REVE-2 was a multicenter study that enrolled 246 patients with anterior wall Q-wave MI from 8 centers in France from February 2006 to September 2008^16^. The research protocol was approved by the ethics committee of the “Centre Hospitalier et Universitaire de Lille” (CP05/91 of December 13^th^ 2005, Lille, France), and written informed consent was obtained from each patient. The protocol required serial echographic studies at hospital discharge (day 3 to day 7) then 3 months and 1 year after MI; serial blood sampling was performed at hospital discharge then 1 month, 3 months, and 1 year after MI. Baseline characteristics of REVE-2 population are summarized in Table 1. REVE-2 patients were divided as no remodeling (LVR < 20%) and high remodeling (LVR > 20%) according to the LV remodeling determined as ((EDV1year − EDV_baseline_)/EDV_baseline_).

### Cell Culture

The rat embryonic-heart derived H9c2 cell line (ATCC, CRL-1446) were used followed manufacturer’s instructions. The human embryonic-heart derived Cytivia Plus Cardiomyocytes (GE Healthcare, 29-0918-80) were used following manufacturer’s instructions. Cells were then treated with Isoproterenol (Iso, 10 µM) in serum-free medium for 24 h and 48 h.

### Luciferase Reporter Assay

Human SOD2 3′UTR (824 bp) harboring two potential binding sites for miR-222-3p was cloned into SpeI and HindIII cloning site of pMIR-REPORT vector (Ambion). The resulting construct (20 ng) was co-transfected with control mirVana mimic or mirVana mimic miR-222-3p (each 30 nM, ThermoFisher Scientific) and 20 ng of β-galactosidase control plasmid (Promega) into 48well-plated HEK293 reporter cells by the use of Lipofectamine 2000 (Invitrogen). Cells were incubated for 24 h before detecting luciferase and β-galactosidase activity applying different substrates (Promega).

### Transfection

#### SOD2 siRNA

The specific siRNA specifically targeting rat SOD2 mRNA (SOD2 siRNA) and non-targeting control (NT siRNA) were used (ON-TARGETplus Rat Sod2 (24787) siRNA - SMARTpool, #L-080048-02-0010, Dharmacon, GE Healthcare). H9c2 were plated (300,000 cells/well) in 6-well plates and were allowed to grow for 24 h without antibiotics. SOD2 or NT siRNA (25 nmol/L) were transfected with the DharmaFECT® reagent (4 µL) according to the manufacturer’s recommendations. Total cell extracts were collected 72 h after transfection.

#### miR-222-3p modulation

The specific miR-222 mimic and miR-222 inhibitor and negative control were used (miRVAna, Life Technologies). Cytivia Plus cardiomyocytes were plated (200,000 cells/well) in 6-well plates and were allowed to grow for 1 week in RPMI supplemented with 2% (v/v) B27. MiR-222 mimic, miR-222 inhibitor and their negative control (100 nmol/L) were transfected with the Lipofectamine 2000 (Life Technologies) reagent (4 µL) in OptiMEM (Life Technologies). Medium was changed for RPMI supplemented with 2% (v/v) B27 6 h after transfection. Cells were treated with Iso 10 µM 24 h after transfection. Total cell extracts were collected 72 h after transfection.

### Statistical analysis

For cell culture and animal model, data are expressed as means ± SEM and analysed with GraphPad software. Data were compared using non parametric Mann–Whitney test for 2 groups’ comparison and using Kruskal-Wallis with Dunn’s multiple comparison test for multiple groups’ comparison. Statistical significance was accepted at a p value < 0.05.

For Human studies, the levels of miRs and SOD2 were log-transformed and compared between high remodelers (>20% change in LVEDV between baseline and 1 year) and non-remodelers (<20% change in LVEDV between baseline and 1 year). We used a logistic regression adjusted for age, sex, and baseline LVEDV. All statistical analyses were performed using the STATA 14.1 software (STATA Corporation, College Station, Texas, USA). For analyzing correlation between the circulating levels of SOD2 and miRNAs, we used the Pearson correlation coefficient test. Statistical significance was assumed at a p value < 0.05.

## Electronic supplementary material

Supplementary data
