# Publisher Correction: MicroRNAs regulating superoxide dismutase 2 are new circulating biomarkers of heart failure

**DOI:** 10.1038/s41598-018-35955-7

**Published:** 2018-11-29

**Authors:** Emilie Dubois-Deruy, Marie Cuvelliez, Jan Fiedler, Henri Charrier, Paul Mulder, Eleonore Hebbar, Angelika Pfanne, Olivia Beseme, Maggy Chwastyniak, Philippe Amouyel, Vincent Richard, Christophe Bauters, Thomas Thum, Florence Pinet

**Affiliations:** 1 INSERM, U1167, FHU-REMOD-HF, Institut Pasteur de Lille, University Lille Nord de France, 59800 Lille, France; 2 Institute of Molecular and Translational Therapeutic Strategies (IMTTS), IFB-Tx, Hannover Medical School, Hannover, Germany; 3 Normandie Univ, UNIROUEN, Inserm U1096, FHU-REMOD-HF, 76000 Rouen, France; 4 Centre Hospitalier Régional et Universitaire de Lille, 59800 Lille, France

Correction to: *Scientific Reports* 10.1038/s41598-017-15011-6, published online 07 November 2017

The original version of this Article contained errors, where references to the Supplementary Figures and Tables were incorrectly linked to the Figures and Tables contained in the main text. As a result,

In the Results section, under the subheading ‘Identification of candidate miRNAs for heart failure’,

“Detailed echocardiographic, hemodynamic and morphometric parameters of sham- and MI-rats are provided (Table 1).”

now reads:

“Detailed echocardiographic, hemodynamic and morphometric parameters of sham- and MI-rats are provided (Supplementary Table 1).”

“Interestingly, SOD2 is regulated by 5 of 13 miRNAs selected by IPA, *i.e*. mir-21-3p, miR-21-5p, miR-23a-3p, miR-145-5p and miR-222-3p (Fig. 1A).”

now reads:

“Interestingly, SOD2 is regulated by 5 of 13 miRNAs selected by IPA, *i.e*. mir-21-3p, miR-21-5p, miR-23a-3p, miR-145-5p and miR-222-3p (Supplementary Fig. 1A).”

“Among the 11 miRNAs quantified, 4 were not modulated after 7 days or 2 months MI: miR-29b-3p, miR-338-3p, miR-133a and miR-483-3p interacting respectively with tropomyosin alpha-1 chain, pyruvate kinase PKM and phosphoglycerate mutase 1 (Fig. 1B–E). Moreover, we observed a significant increase in miR-320a and in miR-377-5p in LV of HF-rats respectively in 7 days and 2 months MI-rats (Fig. 1F–G).”

now reads:

“Among the 11 miRNAs quantified, 4 were not modulated after 7 days or 2 months MI: miR-29b-3p, miR-338-3p, miR-133a and miR-483-3p interacting respectively with tropomyosin alpha-1 chain, pyruvate kinase PKM and phosphoglycerate mutase 1 (Supplementary Fig. 1B–E). Moreover, we observed a significant increase in miR-320a and in miR-377-5p in LV of HF-rats respectively in 7 days and 2 months MI-rats (Supplementary Fig. 1F–G).”

In the Results section, under the subheading ‘Post-transcriptional regulators of SOD2 expression’,

“Expression of miR-145-5p was significantly increased in 2 months-rats compared to 7 days-rats (Fig. 1A). We observed a significantly increased expression of the 4 other miRNAs in LV, at 7 days post-MI for miR-23a-3p (Fig. 1D), at 2 months post-MI for miR-21-5p (Fig. 1D) and miR-21-3p (Fig. 1A) and at both times for miR-222-3p (Fig. 1D).”

now reads

“Expression of miR-145-5p was significantly increased in 2 months-rats compared to 7 days-rats (Supplementary Fig. 1A). We observed a significantly increased expression of the 4 other miRNAs in LV, at 7 days post-MI for miR-23a-3p (Fig. 1D), at 2 months post-MI for miR-21-5p (Fig. 1D) and miR-21-3p (Supplementary Fig. 1A) and at both times for miR-222-3p (Fig. 1D).”

In the Results section, under the subheading ‘Circulating miRNAs interacting with SOD2 as prognostic biomarkers of HF’,

“We also identified direct interaction between miR-222-3p, SOD2 and other molecules in the REVE-2 network at baseline (Fig. 3B, details are provided Fig. 2 and Supplementary Table 3).”

now reads:

“We also identified direct interaction between miR-222-3p, SOD2 and other molecules in the REVE-2 network at baseline (Fig. 3B, details are provided Supplementary Fig. 2 and Supplementary Table 3).”

“Conversely, we observed a significant increase of the 3 miRNAs in patients with high remodeling at 3 months post-MI and no modulation at 1 year post-MI (Fig. 3C, left panel and Fig. 3).”

now reads:

“Conversely, we observed a significant increase of the 3 miRNAs in patients with high remodeling at 3 months post-MI and no modulation at 1 year post-MI (Fig. 3C, left panel and Supplementary Fig. 3).”

“The same information was found for the circulating levels of miR-21-5p (Fig. 3A, right panel)”

now reads:

“The same information was found for the circulating levels of miR-21-5p (Supplementary Fig. 3A, right panel)”

“The same information was found for the circulating levels of miR-21-5p (Fig. 3A, bottom panel) and miR-23a-3p (Fig. 3B, bottom panel).”

now reads:

“The same information was found for the circulating levels of miR-21-5p (Fig. 3A, bottom panel) and miR-23a-3p (Supplementary Fig. 3B, bottom panel).”

In the Methods section, under the subheading ‘Animal models’,

“Haemodynamic and echocardiographic measurements (Table 1) were taken 7 days and 2 months after surgery, followed by heart excision and plasma sampling, as previously described^4,29^.”

now reads:

“Haemodynamic and echocardiographic measurements (Supplementary Table 1) were taken 7 days and 2 months after surgery, followed by heart excision and plasma sampling, as previously described^4,29^.”

These errors have now been corrected in the HTML and PDF versions of this Article, and in the accompanying Supplementary Data file.

