# Supplementary material for: MicroRNAs regulating superoxide dismutase 2 are new circulating biomarkers of heart failure
Source: Sci Rep. 2017 Nov 7;7:14747. doi: 10.1038/s41598-017-15011-6 (PMC5676691; doi:10.1038/s41598-017-15011-6)
Supplement: Supplementary file 1 — Supplementary data [file 41598_2017_15011_MOESM1_ESM.pdf]

MicroRNAs regulating superoxide dismutase 2 are new circulating biomarkers of heart failure

Running title: ***MicroRNAs targeting SOD2 as new biomarkers of HF***

Emilie Dubois-Deruy, PhD[a], Marie Cuvelliez, MSC[a], Jan Fiedler, PhD[b], Henri Charrier, MSC[a], Paul Mulder, PhD[c], Eleonore Hebbar, MD[a], Angelika Pfanne[b], Olivia Beseme, MSC[a], Maggy Chwastyniak, MSC[a], Philippe Amouyel, MD PhD[a,d], Vincent Richard, PhD[c], Christophe Bauters, MD[a,d], Thomas Thum, MD PhD[b], and Florence Pinet, PhD[a,*].

[a]INSERM, U1167, FHU-REMOD-HF, Institut Pasteur de Lille, University Lille Nord de France, 59800 Lille, France

[b]Institute of Molecular and Translational Therapeutic Strategies (IMTTS), IFB-Tx, Hannover Medical School, Hannover, Germany

[c]Normandie Univ, UNIROUEN, Inserm U1096, FHU-REMOD-HF, 76000 Rouen, France

[d]Centre Hospitalier Régional et Universitaire de Lille, 59800 Lille, France

*Corresponding author:

Dr Florence PINET, INSERM U1167-IPL, 1 rue du professeur Calmette, 59019 Lille cedex, France

Tel: (33) 3 20 87 72 15

Fax: (33) 3 20 87 78 94

E-mail: florence.pinet@pasteur-lille.fr

Detailed Material and methods

All methods were carried out in accordance with relevant guidelines and regulations and all experimental protocols were approved by Inserm.

Animal models

All animal experiments were performed according to the Guide for the Care and Use of Laboratory Animals published by the US National Institutes of Health (NIH publication NO1-OD-4-2-139, revised in 2011). Animals were used and experimental protocols performed under the supervision of a person authorized to perform experiments on live animals (F. Pinet: 59-350126 and E. Dubois-Deruy: 59-350253). Approval was granted by the institutional ethics review board (CEEA Nord Pas-de-Calais N°242011, January 2012).

Before surgery, rats were anaesthetized (sodium methohexital, 50 mg/kg IP), while analgesia was administered before (xylazine 5 mg/kg IP) and 1h after surgery (xylazine 50 mg/kg subcutaneously) as described[1]. MI was induced in 10-week-old male Wistar rats (n=63) (Janvier, Le Genest St isle, France) by ligation of the left anterior descending coronary artery[1,2]. Haemodynamic and echocardiographic measurements (Supplementary Table 1) were taken 7 days and 2 months after surgery, followed by heart excision and plasma sampling, as previously described[3,4]. Blood samples were collected in EDTA-treated tubes and then centrifuged for 15 min at 3000 rpm to remove cells and platelets. After centrifugation, the plasma was collected, aliquoted and stored at –80°C.

Human samples

REVE-2 was a multicenter study that enrolled 246 patients with anterior wall Q-wave MI from 8 centers in France from February 2006 to September 2008[5]. Inclusion criteria were hospitalization within 24 hours after symptom onset and at least 3 LV segments of the infarct zone that were akinetic at predischarge echocardiography. Exclusion criteria were inadequate

echographic image quality, life-limiting noncardiac disease, significant valvular disease, or previous Q-wave MI. The research protocol was approved by the ethics committee of the Centre Hospitalier et Universitaire de Lille (CP05/91 of December 13th 2005, Lille, France), and written informed consent was obtained from each patient. All experiments were performed in accordance with relevant guidelines and regulations. The protocol required serial echographic studies at hospital discharge (day 3 to day 7) and 3 months and 1 year after MI; serial blood sampling was performed at hospital discharge (day 3 to day 7) and 1 month, 3 months, and 1 year after MI. Plasma were processed within 2 hours, and samples were divided into aliquots and stored at −80°C. Baseline characteristics of REVE-2 population are summarized in Table 1.

Cell Culture

H9c2 cell line

The rat embryonic-heart derived H9c2 cell line (ATCC, CRL-1446) were seeded at a density 1×10^5 cells/well in 6-well plates at 37°C and 5% CO_2 in Dulbecco's Modified Eagle Medium (Life technologies) supplemented with 10% (v/v) Fetal Bovine Serum (ATCC), 1% penicillin and streptomycin (10,000 U/mL). Cells were then treated with Iso (10 µM) in a DMEM, 1% penicillin and streptomycin without serum for 24h and 48h.

Cytivia Plus Cardiomyocytes

The human embryonic-heart derived Cytivia Plus Cardiomyocytes (GE Healthcare, 29-0918-80) were seeded at a density 2×10^5 cells/well in 6-well plates coated with Matrigel 1:30 in RPMI (Life technologies) supplemented with 2% (v/v) B27 supplement (Life technologies) for 7 days at 37°C under 5% CO_2 atmosphere.

Proteomic and phosphoproteomic analysis

Proteomic and phosphoproteomic analysis were performed as already described [3,6]. Briefly, LV proteins (100-500 µg) from sham- and 2 months MI-rats were analyzed by 2D-electrophoresis and specific staining. Detailed protocols as already been published [3,6].

Bioinformatical analysis

Differentially regulated proteins previously identified by mass spectrometry[3,6]were further analyzed using Ingenuity Pathway Analysis (IPA, www.ingenuity;com, Winter Release 2012, Ingenuity Systems, Mountain View, CA). A protein interaction network was generated as follows: a dataset containing the up- and downregulated proteins, called focus proteins, was uploaded into the IPA tool (Supplementary Table 2). These focus proteins were overlaid onto a global molecular network developed from the information in the Ingenuity Pathways Knowledge Base. Networks of these focus proteins were then algorithmically generated by including as many focus proteins as possible and other nonfocus proteins from the database that are needed to generate the network based on connectivity. Here, we focused our analysis on microRNAs with high predicted and experimentally observed selection.

RNA Extraction and qRT-PCR Analyses

Rat LV samples

RNA was extracted from LV (about 60 mg) and homogenized with 2mL TRI Reagent® (Sigma-Aldrich) in Ultra-Turrax®. To ensure complete dissociation of nucleoprotein complexes, samples were kept 5 min at room temperature before addition of 200 µL of chloroform per ml of TRI Reagent. Samples were then shaked vigorously for 15 sec before centrifugation at 12,000 g for 12 min at 4°C to recuperate RNA in the upper aqueous phase. This phase was transferred to a fresh tube and same volume of 2-propanol was added for 2h at -20°C before centrifugation at 13,000 g for 30 min at 4°C. The RNA pellet was washed by

adding 800µL of ethanol before centrifugation at 12,000 g for 10 min at 4°C. Samples were dried and then solubilised in 40µL of RNAse-free water and store at -80°C.

Rat and Human plasma samples

RNA was extracted from 50µL of plasma with miRNeasy Minikit (Qiagen, #217004), as previously described[7]. As an internal spiked-in control, *Coenorhabditis elegans* miR-39 (Cel-39-1) was added during the isolation process. Samples were dried and then solubilised in 10 µL of RNAse-free water and store at -80°C.

Cell samples

RNA was extracted from H9c2 and Cytivia 6-wells plate with miRNeasy Minikit (Qiagen, #217004). For Cytivia, Matrigel was previously digested by Corning Cell Recovery Solution (#354253) according to the manufacturer's instructions.

RT-qPCR analyses

Quality of LV RNA extraction was checked with Pico600 RNA (Agilent) to determine the RNA Integrity Number (RIN). Reverse-transcription was performed with 100 ng of rat LV and plasma total RNA using the miScript II RT kit (Qiagen) and the cDNA was amplified with QuantiTect® SYBR® Green PCR kit (Qiagen) for micro-RNAs and miScript SYBR Green PCR (Qiagen) for mRNA on a Mx3000P Q-PCR system (Agilent Technologies), according to the manufacturer's instructions.

The human plasma RNA was reverse transcribed with TaqMan MicroRNA Reverse Transcription Kit (Applied Biosystems, #4366597) and specific miRNA Reverse Transcription Primers (Applied Biosystems, #4427975) for miR-21-5p, miR-23a-3p and miR-222-3p and Cel-39-1 as normalization control and RT-PCR analysis was performed using iQSupermix (Biorad, #170-8864) and specific miRNA Taqman Probes (Applied Biosystems, #4427975) using an automatic robotic pipetting device (Agilent).

Protein extraction

Proteins were extracted from 40 mg of LV frozen tissue with Dounce-Potter homogenization or from 6-well plates cell into ice-cold RIPA buffer (50 mmol/L Tris [pH7.4], 150 mmol/L NaCl, 1% Igepal CA-630, 50 mmol/L deoxycholate, and 0.1% SDS) containing anti-proteases (Complete™ EDTA-free, Roche Diagnostics), serine/threonine protein phosphatase inhibitors (Phosphatase inhibitor Cocktail 3, Sigma-Aldrich) and 1 mmol/L Na_3VO_4, as described previously[3]. Lysed cells were incubated for 1h at 4°C, centrifuged 15 min at 12 000 rpm to collect the soluble proteins. Protein concentrations were determined with a Lowry-based method protein assay (Biorad, Marnes-la-Coquette, France) and samples were kept at -80°C.

Western blot

Soluble proteins (5-15 µg) were resolved on 15% SDS-PAGE and transferred on 0.2 µm nitrocellulose membranes (Trans-Blot® Turbo™ Transfert Pack, Bio-Rad). Equal total proteins loads were confirmed by Ponceau red (0.1% Ponceau , Sigma-Aldrich), 5% acetic acid (v/v)] staining of the membranes. The membranes were then blocked in 5% milk in TBS-Tween buffer for 1h before 4°C overnight incubation with SOD2 antibody (Abcam ab16956) or GAPDH (Santa-Cruz, sc-365062, diluted 1/10000 in 5% milk TBS-Tween buffer. Blots were then washed three times with TBS-Tween 0.1% buffer and incubated with mouse horseradish peroxidase-labelled secondary antibodies for 1h (1/10000) in blocking solution. The Chemidoc® camera (Biorad) was used for imaging and densitometry analysis after membranes were incubated with enhanced chemiluminescence (ECL™) western blotting detection reagents (GE Healthcare).

SOD2 ELISA Kit

SOD2 was quantified from 100µL of plasma diluted 1/50 with Human SOD2 ELISA Kit (Abnova), according to the manufacturer's instructions. SOD2 concentration was determinedby average the duplicate readings for each standard, control, and samples and subtract the average zero standard optical density.

Immunofluorescence

Biphotonic confocal microscopy was used for the imaging of 4% paraformaldehyde and 0.1% Triton fixed/permeabilized cardiomyocytes. Immunofluorescence staining was performed by saturation for 30 min with 1% BSA before incubation with the alpha-actinin antibody (sc-7453, Santa-Cruz) overnight at 4°C at dilution 1/50. Alexa Fluor® 568 coupled anti-goat secondary antibody at dilution 1/500 was incubated for 30 min at room temperature before nuclei staining for 10 min at room temperature (Hoechst 33258, Invitrogen™) with mounting medium (H-1500, Vectashield).

Mitochondrial superoxide anion levels were measured using MitoSOX Red (DHE conjugated to a mitochondrial localization tag). Cardiomyocytes were incubated in HBSS with 10 µM MitoSOX Red (Life Technologies) for 20 min at 37°C.

Stainings were visualized with a x40 objective on an LSM710 confocal microscope that used Zen image acquisition and analysis software (Zeiss). Images were acquired with a resolution of at least 1024×1024.

Luciferase Reporter Assay

Human SOD2 3'UTR (824 bp) harboring three potential binding sites for miR-222-3p was cloned into Spe I and Hind III cloning site of pMIR-REPORT vector (Ambion). The resulting construct (20 ng) was co-transfected with control mirVana mimic or mirVana mimic miR-222-3p (each 30nM, ThermoFisher Scientific) and 20 ng of β-galactosidase control plasmid (Promega) into 48 well-plated HEK293 reporter cells by the use of Lipofectamine 2000

(Invitrogen). Cells were incubated for 24h before detecting luciferase and β-galactosidase activity applying different substrates (Promega).

Transfection

SOD2 siRNA

The specific siRNA specifically targeting rat SOD2 mRNA (SOD2 siRNA) and non-targeting control (NT siRNA) were used (ON-TARGETplus Rat Sod2 (24787) siRNA - SMARTpool, #L-080048-02-0010, Dharmacon, GE Healthcare). H9c2were plated (300,000 cells/well) in 6-well plates and were allowed to grow for 24h without antibiotics. SOD2 or NT siRNA (25 nmol/L) were transfected with the DharmaFECT® reagent (4 µL) according to the manufacturer's recommendations. Total cell extracts were collected 72h after transfection.

MiR-222-3p modulation

The specific miR-222 mimic and miR-222 inhibitor and negative control were used (miRVAna, Life Technologies). Cytivia Plus cardiomyocytes were plated (200,000 cells/well) in 6-well plates and were allowed to grow for 1 week in RPMI supplemented with 2% (v/v) B27. miR-222 mimic, miR-222 inhibitor and their negative control (100 nmol/L) were transfected with the Lipofectamine 2000 (Life Technologies) reagent (4 µL) in OptiMEM (Life Technologies). Medium was changed for RPMI supplemented with 2% (v/v) B27 6h after transfection. Cells were treated with Iso 10 µM 24h after transfection. Total cell extracts were collected 72h after transfection.

Molecular data processing

The molecular REVE-2 network model was built based on the molecular data as described[8], including 24 molecular variables (18 proteins, 6 non-coding RNAs (5 miRNAs and 1 long noncodingRNA), measured at 1 to 4 time points (depending on the variables, including baseline, 1 month, 3 months, and 1 year). To build the REVE-2 network model, the

knowledge platform EdgeBox (EdgeLeap's proprietary knowledge platform) was used as a resource of public knowledge on molecular interactions. The REVE-2 network model embeds the REVE-2 molecular variables in an integrated network of known interactions ("edges") between molecules ("nodes"). All visualizations of the REVE-2 network model were performed using Cytoscape, version 3.4.0.

For each node in the network the centrality measure "betweenness" was calculated using the igraph R package. The betweenness of a node is the normalized count of shortest paths between all other nodes in the network passing through that node. High betweenness of a node indicates that a molecule is crucial to maintain functionality and coherence of signaling mechanisms, while a low betweenness indicates a more peripheral role for the molecule.

Statistical analysis

For cell culture and animal model, data are expressed as means ± SEM and analyse with GraphPad software. Data were compared using nonparametric Mann–Whitney test for 2 groups' comparison and using Kruskal-Wallis with Dunn's multiple comparison test for multiple groups' comparison. Statistical significance was accepted at the level of $P<0.05$.

For Human studies, the levels of miRs and SOD2 were log-transformed and compared between high remodelers (>20% change in LVEDV between baseline and 1 year) and non remodelers (<20% change in LVEDV between baseline and 1 year). We used a logistic regression adjusted for age, sex, and baseline LVEDV. All statistical analyses were performed using the STATA 14.1 software (STATA Corporation, College Station, Texas, USA). For analyzing correlation between the circulating levels of SOD2 and miRNAs, we used the Pearson correlation coefficient test. Statistical significance was assumed at a p value <0.05.

References

1. Mulder, P. *et al.* Early versus delayed angiotensin-converting enzyme inhibition in experimental chronic heart failure. Effects on survival, hemodynamics, and cardiovascular remodeling. *Circulation* **95,** 1314–1319 (1997).
2. Pfeffer, M. A, Pfeffer, J. M., Steinberg, C. & Finn, P. Survival after an experimental myocardial infarction: beneficial effects of long-term therapy with captopril. *Circulation* **72,** 406–412 (1985).
3. Dubois, E. *et al.* Decreased Serine207 phosphorylation of troponin T as a biomarker for left ventricular remodelling after myocardial infarction. *Eur. Heart J.* **32,** 115–123 (2011).
4. Mulder, P. *et al.* Long-term heart rate reduction induced by the selective I(f) current inhibitor ivabradine improves left ventricular function and intrinsic myocardial structure in congestive heart failure. *Circulation* **109,** 1674–1679 (2004).
5. Fertin, M. *et al.* Usefulness of serial assessment of B-type natriuretic peptide, troponin I, and C-reactive protein to predict left ventricular remodeling after acute myocardial infarction (from the REVE-2 Study). *Am. J. Cardiol.***106,** 1410–1416 (2010).
6. Cieniewski-Bernard, C. *et al.* Proteomic Analysis of Left Ventricular Remodeling in an Experimental Model of Heart Failure. *J. Proteome Res.***7,** 5004–5016 (2008).
7. Bauters, C. *et al.* Circulating miR-133a and miR-423-5p fail as biomarkers for left ventricular remodeling after myocardial infarction. *Int. J. Cardiol.***168,** 1837–1840 (2013).
8. Pinet, F. *et al.* Integrative network analysis reveals time-dependent molecular events underlying left ventricular remodeling in post-myocardial infarction patients. *BBA-Mol Bas Dis* **1862**, 1445-1453 (2017).

Supplementary Figures

Supplementary Figure 1: MiRNAs expression in LV of HF-rats.

(**A**) Graphical representation of network built from the proteomic data analyzed with IPA focused on SOD2 protein. The full IPA network is described as Supplementary Table 2. Continuous full arrow indicates direct interaction and dotted arrows non-direct interaction. Quantification by RT-qPCR of miR-145-5p (**A**, middle panel), miR-21-3p (**A**, right panel), miR-29b-3p (**B**), miR-338-3p (**C**), miR-133a (**D**), miR-483-3p (**E**), miR-320a (**F**) and miR-377-5p (**G**) in RNA extracted from LV of sham- and HF-rats after 7 days (circle) and 2 months (square) after MI. Data were normalized to miR-423-3p and graph shows mean ± SEM values expressed as fold change ($2^{-\Delta\Delta Ct}$). The protein identified for the interaction with miRNAs by IPA analysis is indicated for each miRNAs. ** p<0.01 from at least 6 LV samples for 2 groups' comparison (nonparametric Mann–Whitney test) and ### p<0.001, and for multiple groups' comparison (Kruskall-Wallis with Dunn's multiple comparison test).

Supplementary Figure 2: Detailed molecular network of miR-222-3p.

Visualization of the REVE-2 molecular network model centralized on the molecules (nodes) interacting with miR-222-3p at baseline (top) and 3 months (bottom) post-MI. MiR-222-3p is circled in red, nodes are colored by cluster membership, based on the 12 clusters that contain REVE-2 variables[8]. Detailed information of the REVE-2 network is provided as Supplementary Table 3.

Supplementary Figure 3: Circulating SOD2 and miRNAs plasma levels of REVE-2 patients.

REVE-2 patients (n=224) were divided as no remodelers (LVR < 20%, n=138) (white) and high remodelers (LVR > 20%, n=86) (grey) according to the LV remodeling determined as $((EDV_{1year} - EDV_{baseline}) / EDV_{baseline})$. (**A**) Quantification by RT-qPCR of miR-21-5p in

plasma extracted from REVE-2 patients at baseline and after 3 months and 1 year post-MI for all the population (left panel). Analysis were also performed by separating women (n=46, 24 no LVR and 20 high LVR) and men (n=200, 114 with no LVR and 66 with LVR) (right panel). Data were expressed as log (miR/cel39) and represented in box plot with the central rectangle for the interquartile range, the segment inside the rectangle for the median and the segment outside for the minimum and maximum (top panels). Pearson correlation analysis between SOD2 and miR-21-5p in plasma levels of REVE-2 patients at 3 month post-MI (bottom panels). *, $p<0.05$ compared to no LVR. The scatter plots show the correlation between SOD2 and miR-222-3p plasma levels of REVE-2 patients at 3 month post-MI for all REVE-2 population (n=246) (left panel) and for men (n=200) (right panel). (**B**) Quantification by RT-qPCR of miR-23a-3p) in plasma extracted from REVE-2 patients at baseline and after 3 months and 1 year post-MI. REVE-2 patients were divided as no remodelers (LVR < 20%) (white) and high remodelers (LVR > 20%) (grey) according to the LV remodeling determined as ((EDV1year – EDVbaseline) / EDVbaseline). for all the population (left panel). Analysis were also performed by separating women (n=46, 24 no LVR and 20 high LVR) and men (n=200, 114 with no LVR and 66 with LVR) (right panel). Data were expressed as log (miR/cel39) and represented in box plot with the central rectangle for the interquartile range, the segment inside the rectangle for the median and the segment outside for the minimum and maximum (top panels). *, $p<0.05$ compared to no LVR. Pearson correlation analysis between SOD2 and miR-23a-3p in plasma levels of REVE-2 patients at 3 month and 1 year post-MI (bottom panels). The scatter plots show the correlation between SOD2 and miR-222-3p plasma levels of REVE-2 patients at 3 month and 1 year post-MI for all REVE-2 population (n=246) (left panels) and for men (n=200) (right panels).

Supplementary Table 1: Echocardiographic, hemodynamic and morphometric parameters in sham and HF-rats.

Parameters	7 days		2 months	
	sham (n=8)	HF (n=7)	sham (n=25)	HF (n=23)
LVEDD, mm			6.7 ± 0.1	10.3 ± 0.3***
LVESD, mm			3.5 ± 0.1	8.7 ± 0.3***
LVEDP, mmHg			1.72 ± 0.41	6.15 ± 1.5**
LVESP, mmHg			126 ± 5	123 ± 3
dP/dt_{max}, 10^{-3} $mmHg.sec^{-1}$			9.9 ± 0.4	7.9 ± 0.4**
$dP/dt_{min,}$ 10-3 mmHg.sec-1			9.5 ± 0.6	6.6 ± 0.5**
FS, %			48 ± 2	17 ± 2***
SV, $mL.beat^{-1}$			0.35 ± 0.01	0.31 ± 0.01*
CO, $mL.min^{-1}$			147 ± 7	123 ± 7*
DPB, mmHg			105 ± 5	95 ± 6
BW, g	319 ± 14	311 ± 7	460 ± 6	459 ± 9
HW, g	0.97 ± 0.02	1.08 ± 0.03*	1.23 ± 0.03	1.58 ± 0.07***
HW/BW, mg/g	3.09 ± 0.19	3.48 ± 0.17	2.69 ± 0.07	3.46 ± 0.16***
RVW, mg	198 ± 8	213 ± 24	210 ± 9	279 ± 23**
LVW, g	0.7 ± 0.02	0.72 ± 0.01	0.92 ± 0.02	1.14 ± 0.05***
AW, mg	66 ± 6.3	143 ± 25.8**	97 ± 8.4	161 ± 18.9**
LW, g	0.93 ± 0.03	1.17 ± 0.09*	1.08 ± 0.02	1.26 ± 0.12

AW, atrial weight; BW, body weight; CO, cardiac output; DPB; diastolic blood pressure; dP/dt_{max}, cardiac contractility; dP/dt_{min}, cardiac relaxation; FS, fractional shortening; HW, heart weight; LVEDD, left ventricle end diastolic diameter; LVEDP, left ventricle end diastolic pressure;LVESD; left ventricle end systolic diameter; LVESP, left ventricle end systolic pressure; LVW, left ventricle weight; LW, lung weight, RVW, right ventricle weight; SV, stroke volume. *, $p<0.05$; ** $p<0.01$; *** $p<0.001$ compared to sham.

Supplementary Table 2: Detailed proteins differentially expressed or phosphorylated in LV of HF-rats overlaid onto a global molecular network in the IPA Knowledge Base.

Accession number	Protein name	Fold-change (HF vs sham)		IPA	
		Expression	Phosphorylation	miRNA identified	predicted interaction
2-oxoglutarate metabolic process (GO:0006103)					
Q6P6R2	Dihydrolipoyl dehydrogenase, mitochondrial	x 2.2	ND		
Q99NA5	Isocitrate dehydrogenase [NAD] subunit alpha, mitochondrial	x 0.62	Induced		
Actin crosslink formation (GO:0051764)					
P50753	Troponin T, cardiac muscle	ND	/ 4.3		
Actin filament capping (GO:0051693)					
P04692	Tropomyosin alpha-1 chain	ND	/ 5.5	miR-29b-3p	Direct
				miR-338-3p	Direct
Activation of signaling protein activity involved in unfolded protein response (GO:0006987)					
P06761	78 kDa glucose-regulated protein	ND	Suppressed		
**Acute-phase response (GO:0006953)**					
P01048	T-kininogen 1	x 3	ND		
Acyl-CoA metabolic process (GO:0006637)					
O55171	Acyl-coenzyme A thioesterase 2, mitochondrial	x 0.62	Induced		
Aerobic respiration (GO:0009060)					
Q68FY0	Cytochrome b-c1 complex subunit 1, mitochondrial	ND	Suppressed		
Age-dependent response to reactive oxygen species (GO:0001315)					
**P07895**	**Superoxide dismutase [Mn], mitochondrial (SOD2)**	**x 2.1**	**ND**	**miR-21-3p**	**Indirect**
				**miR-21-5p**	**Indirect**
				**miR-23a-3p**	**Indirect**
				**miR-145-5p**	**Indirect**
				**miR-222-3p**	**Direct**
Aging (GO:0007568)					
P23928	Alpha-crystallin B chain	x 6.2	/ 4.7		
P15429	Beta-enolase	ND	Suppressed		
P04041	Glutathione peroxidase 1	no	/ 4.6		
P63018	Heat shock cognate 71 kDa protein	ND	x 6.2		
Animal organ regeneration (GO:0031100)					
P67779	Prohibitin	ND	Suppressed		
P11980	Pyruvate kinase PKM	ND	Induced	miR-133a	Indirect

Apopototic mitochondrial changes (GO:0008637)					
Q66HF1	NADH-ubiquinone oxidoreductase 75 kDa subunit, mitochondrial	ND	Suppressed		
Apopototic process (GO:0006915)					
P04797	Glyceraldehyde-3-phosphate dehydrogenase	x 1.6	ND		
ATP metabolic process (GO:0046034)					
P15999	ATP synthase subunit alpha, mitochondrial	x 4.7	Induced		
P10719	ATP synthase subunit beta, mitochondrial	x 2.1	x 4.9		
P31399	ATP synthase subunit d, mitochondrial	x 3.3	/ 1.6		
Carbohydrate metabolic process (GO:0005975)					
P42123	L-lactate dehydrogenase B chain	ND	x 5.1		
O88989	Malate dehydrogenase, cytoplasmic	ND	x 2.9		
P16617	Phosphoglycerate kinase 1	x 0.72	ND		
P48500	Triosephosphate isomerase	x 2	ND		
Cardiac muscle contraction (GO:0060048)					
P16409	Myosin light chain 3	ND	x 12.2		
P08733	Myosin regulatory light chain 2, ventricular/cardiac muscle isoform	ND	Suppressed		
Cellular response to fatty acid (GO:0071398)					
P11884	Aldehyde dehydrogenase, mitochondrial	ND	/ 2.4		
Cellular response to starvation (GO:0009267)					
P02770	Serum albumin	ND	/ 10.5		
Cell redox homeostasis (GO:0045454)					
P35704	Peroxiredoxin-2	x 4	ND	miR-122-5p	Indirect
O35244	Peroxiredoxin-6	x 1.5	Induced	miR-377-5p	Direct
				miR-210	Direct
P04785	Protein disulfide-isomerase	x 3	ND	miR-122-5p	Indirect
Fatty acid beta-oxidation (GO:0006635)					
P14604	Enoyl-CoA hydratase, mitochondrial	x 4.7	ND		
Gluconeogenesis (GO:0006094)					
P25113	Phosphoglycerate mutase 1	x 3.7	ND	miR-483-3p	Direct
Glycolytic process (GO:0006096)					
P04764	Alpha-enolase	ND	x 4.6		

Hydrogen sulfide biosynthetic process (GO:0070814)					
P97532	3-mercaptopyruvate sulfurtransferase	ND	x 12.8		
Intermediate filament organization (GO:0045109)					
P48675	Desmin	ND	x 5		
Mesenchyme migration (GO:0090131)					
P63269	Actin, gamma-enteric smooth muscle	ND	x 19.2		
Mitochondrial electron transport, NADH to ubiquinone (GO:0006120)					
Q68FT1	Ubiquinone biosynthesis protein COQ9, mitochondrial	ND	Suppressed		
Phosphocreatine biosynthetic process (GO:0046314)					
P00564	Creatine kinase M-type	ND	Induced		
Proteasome-mediated ubiquitin-dependent protein catabolic process (GO:0043161)					
P40112	Proteasome subunit beta type-3	x 2.7	ND		
Protein deubiquitination (GO:0016579)					
Q91Y78	Ubiquitin carboxyl-terminal hydrolase isozyme L3	x 2.9	ND		
Regulation of muscle contraction (GO:0006937)					
P97541	Heat shock protein beta-6	x 1.5	ND	miR-320a	Indirect
Response to heat (GO:0009408)					
O35878	Heat shock protein beta-2	No	ND		
Q9QUK5	Heat shock protein beta-7	x 0.21	ND		
Serine-type endopeptidase inhibitor activity (GO:0004867)					
P05545	Serine protease inhibitor A3K	x 2.2	x 5.9		

Accession number corresponded to Uniprot Knowledge base (UniProtKB, release of April 2014). Proteins were classified by first biological process in Gene Ontology (GO). ND: not detected. IPA: Ingenuity Pathway analysis (Winter release 2012).

Supplementary Table 3: Detailed list of components of REVE-2 molecular networks centralized on miR-222-3p identified by Cytoscape analysis[8].

Description	Name	Betweenness	Degree	Cluster
collagen, type V, alpha 2	COL5A2	0.0144	49	cluster 3
FBJ murine osteosarcoma viral oncogene homolog	FOS	0.013	76	cluster 1
**estrogen receptor 1**	**ESR1**	**0.0096**	**54**	**cluster 22**
tumor protein p53	TP53	0.009	59	cluster 19
amyloid beta (A4) precursor protein	APP	0.0088	59	cluster 6
phosphatase and tensin homolog	PTEN	0.0085	27	cluster 4
matrix metallopeptidase 1 (interstitial collagenase)	MMP1	0.0076	35	cluster 9
SWI/SNF related, matrix associated, actin dependent regulator of chromatin, subfamily a, member 4	SMARCA4	0.0054	31	cluster 1
zinc finger and BTB domain containing 7A	ZBTB7A	0.0052	30	no cluster
heat shock protein 90kDa alpha (cytosolic), class A member 1	HSP90AA1	0.0045	42	cluster 15
actin, beta	ACTB	0.0036	42	cluster 4
v-ets avian erythroblastosis virus E26 oncogene homolog 1	ETS1	0.0036	32	cluster 1
intercellular adhesion molecule 1	ICAM1	0.0034	16	cluster 25
TIMP metallopeptidase inhibitor 3	TIMP3	0.0029	6	cluster 2
actin gamma 1	ACTG1	0.0029	27	cluster 4
signal transducer and activator of transcription 5A	STAT5A	0.0027	36	cluster 1
pyruvate kinase, muscle	PKM	0.0027	9	cluster 10
forkhead box O3	FOXO3	0.0025	23	cluster 1
host cell factor C1	HCFC1	0.0023	18	cluster 1
ribosomal protein L12	RPL12	0.0023	42	cluster 8
eukaryotic translation initiation factor 2, subunit 1 alpha, 35kDa	EIF2S1	0.0022	22	cluster 8
death-domain associated protein	DAXX	0.002	14	cluster 14
notchless homolog 1 (Drosophila)	NLE1	0.0019	22	cluster 1
trinucleotide repeat containing 6B	TNRC6B	0.0018	10	cluster 39
heterogeneous nuclear ribonucleoprotein H1 (H)	HNRNPH1	0.0018	16	cluster 18
talin 1	TLN1	0.0016	23	cluster 4
dystroglycan 1 (dystrophin-associated glycoprotein 1)	DAG1	0.0016	8	no cluster
splicing factor 3b, subunit 3, 130kDa	SF3B3	0.0015	15	cluster 18
karyopherin (importin) beta 1	KPNB1	0.0015	15	cluster 33
valosin containing protein	VCP	0.0014	13	cluster 32
minichromosome maintenance complex component 3	MCM3	0.0014	14	cluster 12
minichromosome maintenance complex component 7	MCM7	0.0014	14	cluster 12
ribosomal protein S2	RPS2	0.0014	32	cluster 8
interleukin-1 receptor-associated kinase 1	IRAK1	0.0013	18	cluster 21
tumor necrosis factor (ligand) superfamily, member 10	TNFSF10	0.0013	17	cluster 14
cyclin-dependent kinase inhibitor 1B (p27, Kip1)	CDKN1B	0.0013	24	cluster 12
protein phosphatase 2, regulatory subunit A, alpha	PPP2R1A	0.0013	33	cluster 8
eukaryotic translation initiation factor 3, subunit B	EIF3B	0.0012	27	cluster 8
ATPase, class VI, type 11B	ATP11B	0.0012	6	cluster 10
reversion-inducing-cysteine-rich protein with kazal motifs	RECK	0.0011	4	cluster 2
autism susceptibility candidate 2	AUTS2	0.0011	6	cluster 1
excision repair cross-complementation group 6-like	ERCC6L	0.0011	14	cluster 15
filamin A, alpha	FLNA	0.0011	10	cluster 4
SEC24 family member C	SEC24C	0.001	8	no cluster
hes family bHLH transcription factor 1	HES1	0.001	7	cluster 37
KIT ligand	KITLG	0.001	14	cluster 4
family with sequence similarity 126, member B	FAM126B	0.001	4	cluster 2
zinc finger, FYVE domain containing 16	ZFYVE16	0.001	5	cluster 28
growth factor receptor-bound protein 10	GRB10	0.0009	14	cluster 4
DEAD (Asp-Glu-Ala-Asp) box helicase 21	DDX21	0.0009	8	cluster 10
proteasome (prosome, macropain) 26S subunit, ATPase, 4	PSMC4	0.0009	10	cluster 12
ribosomal protein L8	RPL8	0.0009	30	cluster 8
high mobility group AT-hook 1	HMGA1	0.0009	14	cluster 1
cell cycle associated protein 1	CAPRIN1	0.0009	3	cluster 2
spectrin, beta, non-erythrocytic 2	SPTBN2	0.0008	9	no cluster
coronin, actin binding protein, 1A	CORO1A	0.0008	20	cluster 1
phosphatidylinositol-4-phosphate 5-kinase, type I, alpha	PIP5K1A	0.0008	11	cluster 4
pericentrin	PCNT	0.0008	16	cluster 15
transmembrane protein 245	TMEM245	0.0008	2	cluster 5
ezrin	EZR	0.0008	13	cluster 14
dicer 1, ribonuclease type III	DICER1	0.0008	9	cluster 39
vinculin	VCL	0.0008	16	cluster 4

far upstream element (FUSE) binding protein 1	FUBP1	0.0008	3	cluster 5
intracisternal A particle-promoted polypeptide	IPP	0.0008	3	cluster 12
ALG3, alpha-1,3- mannosyltransferase	ALG3	0.0008	9	cluster 1
dedicator of cytokinesis 5	DOCK5	0.0008	3	cluster 12
v-kit Hardy-Zuckerman 4 feline sarcoma viral oncogene homolog	KIT	0.0007	16	cluster 4
transmembrane emp24 protein transport domain containing 7	TMED7	0.0007	12	cluster 14
nucleolar and coiled-body phosphoprotein 1	NOLC1	0.0007	5	no cluster
enolase 1, (alpha)	ENO1	0.0006	11	cluster 1
tyrosine 3-monooxygenase/tryptophan 5-monooxygenase activation protein, gamma	YWHAG	0.0006	19	cluster 15
ubiquitin specific peptidase 15	USP15	0.0006	10	cluster 28
zinc finger protein 460	ZNF460	0.0006	14	cluster 1
kinesin family member 4A	KIF4A	0.0006	7	no cluster
homeodomain interacting protein kinase 2	HIPK2	0.0006	5	cluster 10
QKI, KH domain containing, RNA binding	QKI	0.0006	16	cluster 1
general transcription factor IIIC, polypeptide 5, 63kDa	GTF3C5	0.0006	5	no cluster
lectin, galactoside-binding, soluble, 3 binding protein	LGALS3BP	0.0006	3	no cluster
eukaryotic translation elongation factor 2	EEF2	0.0005	18	cluster 8
transcription elongation factor B (SIII), polypeptide 2 (18kDa, elongin B)	TCEB2	0.0005	6	no cluster
interferon regulatory factor 2 binding protein 2	IRF2BP2	0.0005	10	cluster 1
replication factor C (activator 1) 1, 145kDa	RFC1	0.0005	10	cluster 32
GDP dissociation inhibitor 1	GDI1	0.0005	9	cluster 29
pellino E3 ubiquitin protein ligase family member 2	PELI2	0.0005	8	cluster 21
integrin-linked kinase	ILK	0.0005	10	cluster 4
centrosomal protein 250kDa	CEP250	0.0005	14	cluster 15
cyclin-dependent kinase inhibitor 1C (p57, Kip2)	CDKN1C	0.0005	7	cluster 22
protein phosphatase 2, regulatory subunit B, alpha	PPP2R2A	0.0005	14	cluster 8
isoleucyl-tRNA synthetase	IARS	0.0005	10	no cluster
ubiquitin specific peptidase 9, X-linked	USP9X	0.0004	7	cluster 1
BCL2 binding component 3	BBC3	0.0004	11	cluster 19
proline-rich coiled-coil 2A	PRRC2A	0.0004	3	cluster 5
zw 10 kinetochore protein	ZW10	0.0004	10	cluster 15
exosome component 1	EXOSC1	0.0004	7	cluster 8
ceramide synthase 2	CERS2	0.0004	3	no cluster
Obg-like ATPase 1	OLA1	0.0004	9	cluster 1
translocase of inner mitochondrial membrane 50 homolog (S. cerevisiae)	TIMM50	0.0004	4	cluster 30
misshapen-like kinase 1	MINK1	0.0004	8	cluster 10
acetyl-CoA carboxylase alpha	ACACA	0.0004	6	cluster 10
poly(rC) binding protein 2	PCBP2	0.0004	12	cluster 18
MYC-associated zinc finger protein (purine-binding transcription factor)	MAZ	0.0004	4	cluster 16
drebrin-like	DBNL	0.0004	5	cluster 26
GDP dissociation inhibitor 2	GDI2	0.0004	9	cluster 29
transmembrane and ubiquitin-like domain containing 1	TMUB1	0.0004	4	no cluster
cyclin-dependent kinase 18	CDK18	0.0004	5	cluster 10
topoisomerase (DNA) III alpha	TOP3A	0.0003	4	cluster 32
pantothenate kinase 3	PANK3	0.0003	5	cluster 10
thioredoxin	TXN	0.0003	7	cluster 33
chromosome 8 open reading frame 33	C8orf33	0.0003	4	cluster 5
bromodomain PHD finger transcription factor	BPTF	0.0003	5	no cluster
metastasis associated 1 family, member 2	MTA2	0.0003	7	no cluster
testis expressed 10	TEX10	0.0003	8	cluster 1
low density lipoprotein receptor-related protein 10	LRP10	0.0003	5	cluster 19
ubiquitin-like modifier activating enzyme 1	UBA1	0.0003	7	cluster 1
ribosomal protein S17	RPS17	0.0003	24	cluster 8
CASK interacting protein 2	CASKIN2	0.0003	6	no cluster
phosphorylated adaptor for RNA export	PHAX	0.0003	3	no cluster
heterogeneous nuclear ribonucleoprotein D (AU-rich element RNA binding protein 1, 37kDa)	HNRNPD	0.0003	12	cluster 18
PAN2 poly(A) specific ribonuclease subunit	PAN2	0.0003	5	cluster 8
malate dehydrogenase 2, NAD (mitochondrial)	MDH2	0.0003	5	cluster 35
profilin 1	PFN1	0.0003	8	cluster 1

selectin E	SELE	0.0003	5	cluster 5
zinc finger, FYVE domain containing 9	ZFYVE9	0.0003	5	cluster 28
insulin receptor substrate 4	IRS4	0.0003	9	cluster 4
chondrosarcoma associated gene 1	CSAG1	0.0003	11	cluster 1
erythrocyte membrane protein band 4.1-like 2	EPB41L2	0.0003	10	cluster 1
ATPase, Cu++ transporting, beta polypeptide	ATP7B	0.0003	5	cluster 10
importin 5	IPO5	0.0003	5	cluster 33
cancer susceptibility candidate 3	CASC3	0.0003	18	cluster 8
mortality factor 4 like 1	MORF4L1	0.0003	8	no cluster
claudin 23	CLDN23	0.0002	2	cluster 16
serine/arginine repetitive matrix 2	SRRM2	0.0002	4	cluster 5
pre-B-cell leukemia homeobox 2	PBX2	0.0002	3	no cluster
protein phosphatase 6, catalytic subunit	PPP6C	0.0002	3	cluster 20
eukaryotic translation initiation factor 3, subunit I	EIF3I	0.0002	19	cluster 8
HAUS augmin-like complex, subunit 8	HAUS8	0.0002	4	cluster 1
parkinson protein 7	PARK7	0.0002	3	cluster 5
microtubule-actin crosslinking factor 1	MACF1	0.0002	3	cluster 13
protein phosphatase, Mg2+/Mn2+ dependent, 1H	PPM1H	0.0002	3	cluster 20
ataxin 7-like 3	ATXN7L3	0.0002	3	cluster 37
methionyl-tRNA synthetase	MARS	0.0002	5	no cluster
RAN binding protein 10	RANBP10	0.0002	5	cluster 22
mesoderm development candidate 2	MESDC2	0.0002	3	cluster 2
exosome component 10	EXOSC10	0.0002	4	cluster 33
non-SMC condensin I complex, subunit D2	NCAPD2	0.0002	3	cluster 1
calcium binding tyrosine-(Y)-phosphorylation regulated	CABYR	0.0002	2	cluster 16
**superoxide dismutase 2, mitochondrial**	**SOD2**	**0.0002**	**5**	**cluster 1**
SAP30-like	SAP30L	0.0002	3	no cluster
calpastatin	CAST	0.0002	3	cluster 16
kinesin family member 3B	KIF3B	0.0002	4	no cluster
synaptic vesicle glycoprotein 2A	SV2A	0.0002	2	cluster 16
plexin B2	PLXNB2	0.0002	4	cluster 1
casein kinase 1, gamma 1	CSNK1G1	0.0002	4	cluster 10
slingshot protein phosphatase 2	SSH2	0.0002	3	cluster 20
fatty acid synthase	FASN	0.0002	7	cluster 5
chromobox homolog 2	CBX2	0.0002	3	no cluster
folylpolyglutamate synthase	FPGS	0.0002	4	cluster 10
phosphatidylethanolamine binding protein 1	PEBP1	0.0002	4	cluster 16
oxysterol binding protein-like 10	OSBPL10	0.0002	8	cluster 1
ubiquitin-like with PHD and ring finger domains 2, E3 ubiquitin protein ligase	UHRF2	0.0002	4	cluster 13
Sad1 and UNC84 domain containing 2	SUN2	0.0002	6	cluster 1
polymerase (DNA directed), delta 2, accessory subunit	POLD2	0.0002	4	cluster 32
poly(A) binding protein interacting protein 2	PAIP2	0.0002	3	no cluster
UbiA prenyltransferase domain containing 1	UBIAD1	0.0001	2	no cluster
POM121 transmembrane nucleoporin	POM121	0.0001	5	cluster 33
maestro heat-like repeat family member 1	MROH1	0.0001	2	cluster 13
adaptor-related protein complex 1, beta 1 subunit	AP1B1	0.0001	3	no cluster
sortilin 1	SORT1	0.0001	4	cluster 1
lysine (K)-specific methyltransferase 2D	KMT2D	0.0001	3	cluster 5
pyridoxal-dependent decarboxylase domain containing 1	PDXDC1	0.0001	2	cluster 5
deoxyribose-phosphate aldolase (putative)	DERA	0.0001	2	cluster 5
SERTA domain containing 4	SERTAD4	0.0001	2	cluster 5
kinesin family member 5C	KIF5C	0.0001	4	cluster 5
cytoskeleton associated protein 2	CKAP2	0.0001	2	cluster 5
LYN proto-oncogene, Src family tyrosine kinase	LYN	0.0001	3	cluster 21
YY1 associated protein 1	YY1AP1	0.0001	3	cluster 5
microtubule-associated protein 1B	MAP1B	0.0001	3	no cluster
ring finger protein 215	RNF215	0.0001	4	cluster 1
cyclin-dependent kinase-like 5	CDKL5	0.0001	3	cluster 10

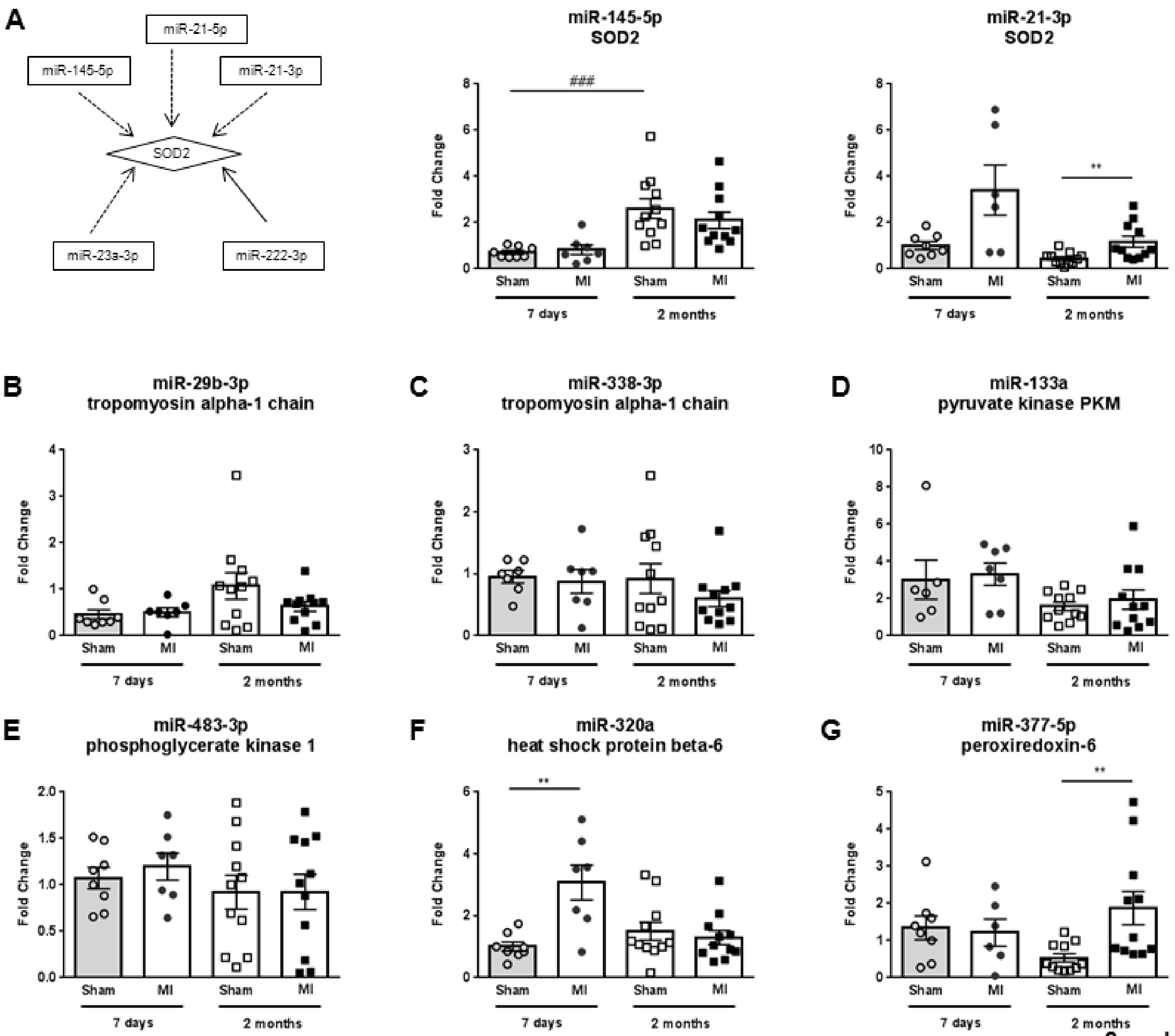

Supplementary Figure 1

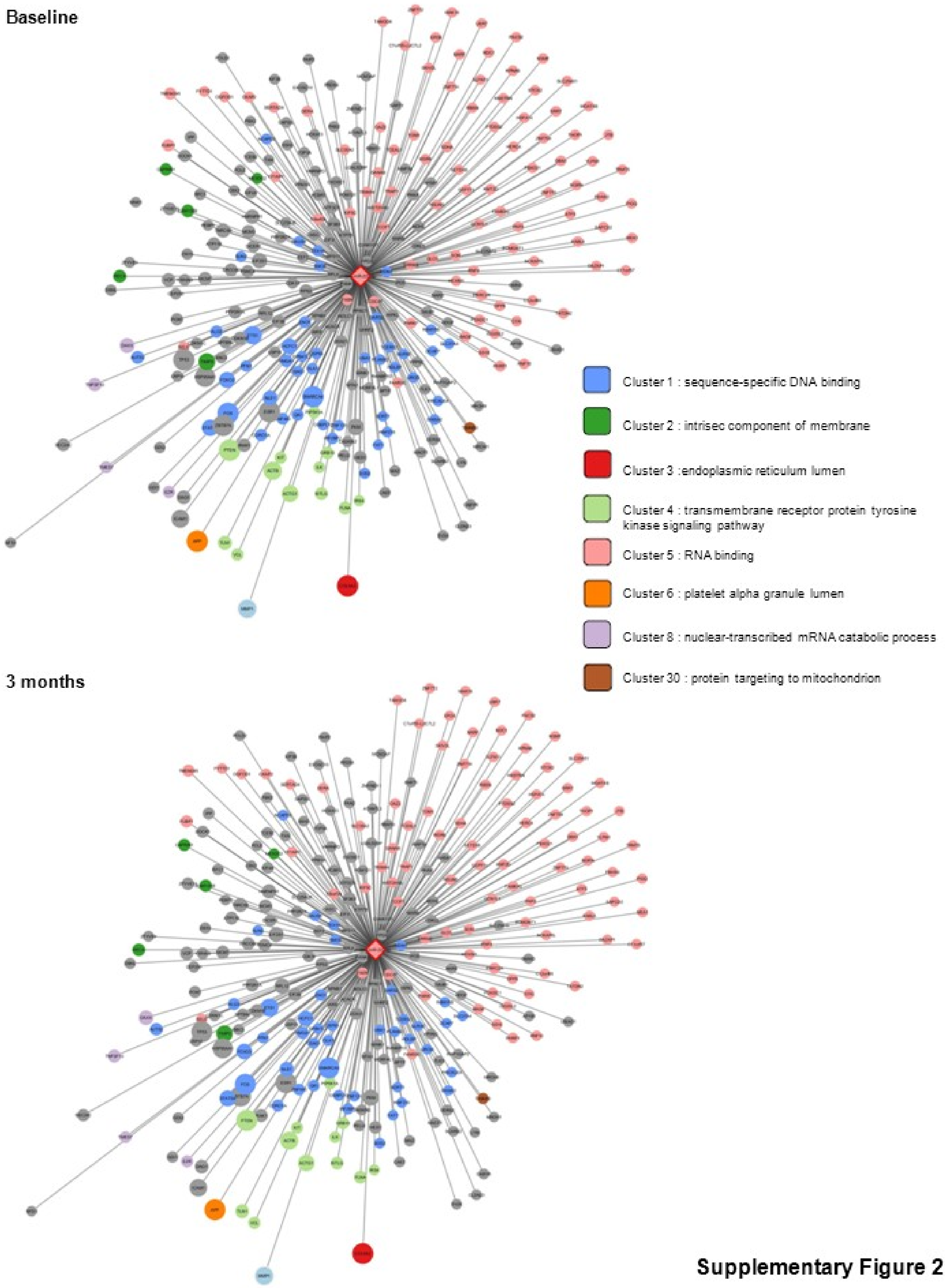

Supplementary Figure 2

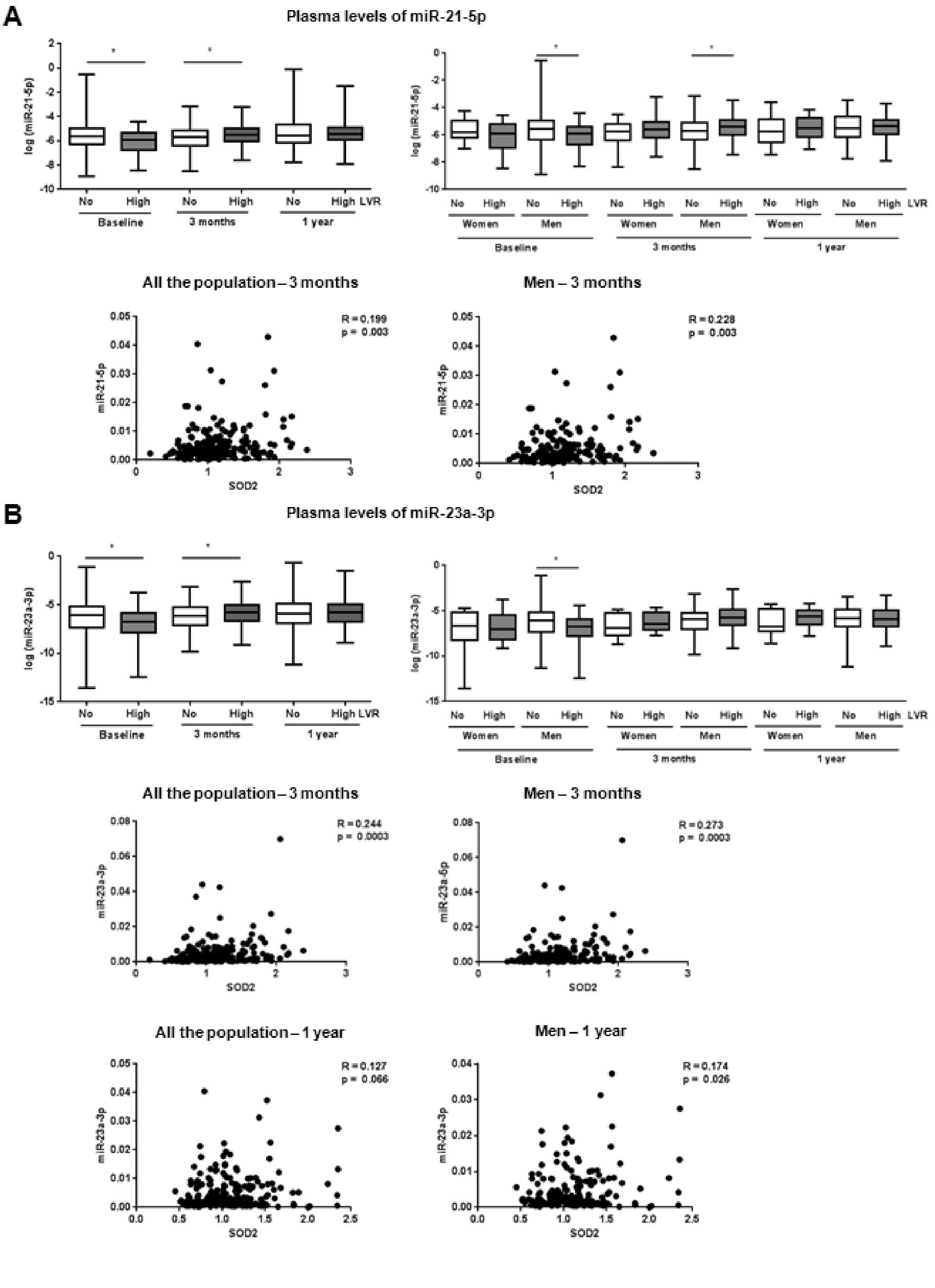

Supplementary Figure 3
